# PARP9-PARP13-PARP14 axis tunes colorectal cancer response to radiotherapy

**DOI:** 10.1186/s13046-025-03439-y

**Published:** 2025-07-11

**Authors:** Rimvile Prokarenkaite, Karolina Kuodyte, Greta Gudoityte, Elzbieta Budginaite, Daniel Naumovas, Egle Strainiene, Kristijonas Velickevicius, Audrius Dulskas, Ernestas Sileika, Jonas Venius, Virginijus Tunaitis, Augustas Pivoriunas, Vytaute Starkuviene, Vaidotas Stankevicius, Kestutis Suziedelis

**Affiliations:** 1 Laboratory of Molecular Oncology, National Cancer Institute, Vilnius, Lithuania; 2 Institute of Biosciences, Life Sciences Center, Vilnius University, Vilnius, Lithuania; 3 Institute of Biotechnology, Life Sciences Center, Vilnius University, Vilnius, Lithuania; 4 Max Planck Institute of Molecular Cell Biology and Genetics, Dresden, Germany; 5 Department of Oncology-Pathology, Science for Life Laboratory, Karolinska Institutet, Solna, Sweden; 6 Department of Pathology, GROW - Research Institute for Oncology and Reproduction, Maastricht University Medical Center+, Maastricht, The Netherlands; 7 Department of Precision Medicine, GROW - Research Institute for Oncology and Reproduction, Maastricht University Medical Center+, Maastricht, The Netherlands; 8 Vilnius Santaros Klinikos Biobank, Vilnius University Hospital Santaros Klinikos, Vilnius, Lithuania; 9 Department of Chemistry and Bioengineering, Vilnius Gediminas Technical University, Vilnius, Lithuania; 10 Institute of Clinical Medicine, Faculty of Medicine, Vilnius University, Vilnius, Lithuania; 11 Department of Surgical Oncology, National Cancer Institute, Vilnius, Lithuania; 12 Department of Radiotherapy, National Cancer Institute, Vilnius, Lithuania; 13 Medical Physics Department, National Cancer Institute, Vilnius, Lithuania; 14 Biomedical physics laboratory, National Cancer Institute, Vilnius, Lithuania; 15 Department of Stem Cell Biology, State Research Institute Centre for Innovative Medicine, Vilnius, Lithuania; 16 BioQuant, Heidelberg University, Heidelberg, Germany

**Keywords:** 3D cell culture, *PARP9*, *PARP13*, *PARP14*, Interferon stimulated genes, Radiotherapy, Radiosensitizers

## Abstract

**Background:**

Colorectal cancer (CRC) is the third most prevalent cancer worldwide. Despite substantial advancements in CRC therapy in recent years, ionizing radiation (IR) continues to be the predominant treatment for colon malignances. However, it still lacks the precision required for excellent therapeutic outcomes, ultimately resulting in tumor radioresistance. This study seeks to explore the potential of atypical PARPs including *PARP9*, *PARP12*, *PARP13* and *PARP14* as innovative radiosensitizing targets for CRC.

**Methods:**

We utilized CRISPR/Cas9-mediated gene editing to knockout the *PARP9*, *PARP12*, *PARP13* and *PARP14* in HT29 and DLD1 cells. The cells were exposed to either a single dose of 6–10 Gy or to fractionated dose of 5 × 2 Gy X-ray radiation cultivating cells in 2D, laminin-rich ECM 3D and multicellular spheroid models. The transcriptomes of nonirradiated and irradiated cells were analyzed using microarrays. Gene set enrichment analysis was conducted to determine the pathways in which *PARP13* is engaged. Cell viability was assessed using a clonogenic assay. Gene expression levels in cells and patient samples were quantified using RT-qPCR.

**Results:**

The expression of *PARP9*, *PARP12*,* PARP13* and *PARP14* was particularly elevated in irradiated colorectal cancer HT29 cells in a microenvironment-dependent manner. *PARP13* deficiency significantly enhanced the sensitivity of HT29 cells to both single-dose and multifractionated irradiation regimens, resulting in reduced colony formation and spheroidal integrity. Microarray analysis indicated that *PARP13* may modulate the expression genes associated with immune response signaling pathways, including members of PARP family. Furthermore, *PARP13* loss in HT29 cells markedly impaired the expression of immune response related genes following multifractionated ionizing irradiation. Finally, chemoradiotherapy significantly elevated the expression of *PARP9*, *PARP12*, *PARP13* and *PARP14* in rectal tumors, while having no effect on adjacent normal colon tissues. Elevated pre-treatment *PARP9* expression levels and a blunted post-treatment increase in *PARP9* and *PARP14* expression predicted poor overall survival in rectal cancer patients, while *PARP13* emerged as the most significant discriminator between tumor and healthy tissue.

**Conclusions:**

Collectively, the *PARP9*/13/14 axis is implicated in the response of CRC to radiation treatment in both preclinical and clinical settings, demonstrating the atypical members of the PARP family as attractive targets for neoadjuvant radiotherapy.

**Supplementary Information:**

The online version contains supplementary material available at 10.1186/s13046-025-03439-y.

## Background

Colorectal cancer (CRC), a prevalent malignancy of the colon and rectum, ranks as the third most common cancer globally and is the second leading cause of cancer-related deaths [1]. However, colorectal cancer is often diagnosed as Stage II or above when treatment often necessitates a combination of chemoradiotherapy and surgical treatment [2]. While CRC therapy has made significant progress in recent years, ionizing radiation (IR) remains a current standard therapy for colon malignancies [3]. IR induces DNA damage in tumor cells, including abasic lesions, deoxyribose ring opening, single and double-stranded breaks (SSBs and DSBs), ultimately leading to cell death [4]. Conversely, tumor cell resistance to IR hinges on their ability to trigger a cascade of cellular responses as a part of DNA damage response (DDR) and a successful DNA damage repair [5]. Advancements in radiotherapy equipment and treatment planning enable the shaping of radiation dose to target tumors while minimizing exposure to surrounding healthy tissues. Despite its potency, radiotherapy can lack the precision required for optimal therapeutic outcomes, ultimately leading to tumor radioresistance. To address this challenge, the discovery of novel biological therapy targets that could enhance damage to cancer cells while significantly sparing normal tissue cells remains increasingly important.

Among the key players in the DDR networks are members of the 17 PARP (poly-ADP-ribose polymerase) protein family. These proteins typically exhibit ADP-ribosylation activity, transferring nicotinamide adenine dinucleotide (NAD^+^) to target molecules. Beyond their involvement in various biological processes - such as transcription control, cell death, and inflammation [6]– some PARPs specifically contribute to attracting repair proteins to sites of DNA damage [7]. PARP1, the most well-studied member, acts as a DNA damage sensor and signal transducer synthesizing poly-ADP-ribose chains that recruit DNA repair factors and remodel chromatin structure around damaged DNA [8]. Consequently, targeting PARP activity has gained prominence in cancer therapy, as the first synthetic lethal targeted therapy. Successful inhibition of PARP1 activity leads to unrepaired SSB accumulation, followed by DSB generation, therefore combining PARP inhibitors with treatment targeting tumors possessing defective DDR systems hold the potential for creating effective combination therapy strategies [9]. Notably, recent studies have highlighted that PARP inhibitors could sensitize tumors to radiotherapy [10–12].

In addition to well-characterized PARPs, other members of this family hold promise for cancer therapeutic applications as they also take part in DNA damage response [13, 14]. For instance, *PARP13* scaffolds PARP1-HSF1 complex at DNA break sites to recruit DNA repair machinery [13], while *PARP14* is a critical factor in stabilizing stalled replication forks, particularly in cells with BRCA1/2 deficiencies [14]. Moreover, lack of *PARP14* impairs homologous recombination-mediated repair of double-strand breaks, resulting in increased sensitivity to DNA damaging agents [15, 16]. *PARP9*, recruited to DNA damage sites, forms a complex with DTX3L, contributing to genome integrity through targeted ubiquitination of p53 [17]. Meanwhile, the precise role of *PARP12* in DNA damage repair remains to be fully elucidated, its interaction with PARP1 suggests a potential involvement in the process [18, 19]. Therefore, the initial evidence on lesser-explored PARP family members indicates that these proteins may serve as promising targets for radiosensitization and could open a potential window for the application of adjuvant therapy following radiotherapy.

While two-dimensional (2D) monolayer cell culture models have been invaluable in cancer research, they often fall short in capturing the complexities of the tumor microenvironment. Three-dimensional (3D) cell cultures, however, offer a more accurate representation of in vivo cancer tissue by mimicking the spatial architecture, nutrient gradients, and cell-cell interactions observed with solid tumors [20]. In addition, the tumor microenvironment modulates cancer immunity features, influences cell shape, proliferation, gene expression and response to therapy [21]. As a result, to enhance comprehension of the complexities of tumor response to treatment and to propose innovative paths for cancer therapy development, the utilization of 3D cell culture models is indispensable.

We previously discovered that the multifractionated dose IR upregulated the expression of unconventional PARP family members including *PARP9*, *PARP12*, *PARP13* and *PARP14* in colorectal cancer cells grown in laminin-rich ECM 3D cell culture [22]. Currently, there is no consolidated data on how *PARP9*, *PARP12*, *PARP13* and *PARP14* affect the response to ionizing radiation in cancer cells. Consequently, this study exploited comprehensive transcriptome analysis to evaluate the potential application of these atypical PARPs as radiosensitizers for colorectal cancer cells in 3D cell culture models. Finally, we conclude that selected PARPs are involved in the response of CRC to radiotherapy, indicating that these proteins are promising targets for the improvement of future treatment options.

## Methods

### Cell line maintenance

Human normal colon tissue CRL1790 and colorectal carcinoma DLD1, HT29, HCT116 and SW48 cell lines were obtained from the American Type Culture Collection (Rockville, Maryland, USA). Cells were maintained in RPMI-1640 (DLD1, HCT116) or DMEM (CRL1790, HT29, SW48) cell culture media (Thermo Scientific, USA) supplemented with 10% fetal bovine serum (Biochrom, Germany), 2 mM glutamine (Thermo Scientific), 100 U/ml penicillin (Carl Roth, Germany) and 0,1 mg/ml streptomycin (Carl Roth, Germany). Cells were incubated at 37 °C in a humidified atmosphere containing 5% CO_2_.

### Cell culture models

For the 2D cell culture, CRL1790, DLD1, HT29, HCT116 and SW48 cells were plated in T25 flasks (30000 cells/cm^2^). For lr-ECM 2D cell culture model, 25,000 CRL1790 cells were embedded in 3.2% lr-ECM protein mixture Geltrex (Thermo Scientific) in DMEM mixture and seeded into 24 well plates pre-coated with 10% Geltrex in DMEM mixture. For lr-ECM 3D cell culture, 75,000 of DLD1 and HT29 cells were embedded in 3.2% Geltrex in culture media in 24 well plates as described previously [23]. Multicellular spheroids (MCS) were formed using liquid-overlay technique as described previously [24]. Briefly, cells were suspended in 200 µl cell culture medium and plated in each well of 96 U-bottom well plates and centrifuged at 1000 g for 10 min. To avoid cell attachment to the well bottom, each well was pre-coated with 1% agarose in sterile water. Cell number established empirically for each cell line to form 400 ± 20 μm size spheroids 2 days after plating. Cells were photographed every two days with inverted optical microscope Eclipse TS100 using digital camera DS-Fi2 (Nikon, Japan).

### Patient samples

The institutional review board approved the study. Patients diagnosed with rectal cancer received neoadjuvant long-course chemoradiotherapy which included 25–28 fractions of irradiation (total dose of 45–51 Gy) and fluorouracil-based treatment during a 5-week period. Tumor and adjacent normal rectal tissue samples were obtained by biopsy and tumor resection surgery 8 or 12 weeks after the neoadjuvant treatment and stored at − 80 °C in RNAlater (Thermo Scientific) until needed. The sample cohort contained four groups and included rectal tumor (*n* = 67), and adjacent normal (*n* = 31) tissue samples collected from patients before long course neoadjuvant treatment (biopsies) and samples collected from the same patients after the therapy (during the surgery). Patient demographic and clinical characteristics are summarized in Table 1.

**Table 1:** Demographic and clinicopathological characteristics of rectal cancer patients

Factor		Total	%
Age (median, range)		68 (41–87)	
Sex	Male	36	53.7
	Female	31	46.3
Stage	1	2	3
	2	5	7.5
	3	59	88.1
	4	1	1.5
Pretreatment staging			
cT	2	5	7.5
	3	51	76.1
	4	11	16.4
cN	0	4	6
	1	16	23.9
	2	47	70.1
Pathological staging			
ypT	0	8	11.9
	1	2	3
	2	17	25.4
	3	35	52.2
	4	5	7.5
ypN	0	43	64.2
	1	17	25.4
	2	7	10.4
Dworak Tumor Regression Grading	1	19	28.4
	2	31	46.3
	3	9	13.4
	4	8	11.9
Interval between neoadjuvant chemoradiation completion and surgery (weeks)	8	30	44.8
	12	37	55.2

### CRISPR/Cas9 editing and clone selection

sgRNAs were designed using Benchling CRISPR sgRNA Design tool [25]. sgRNAs sequences are shown in Additional Table 1. For transient expression of the CRISPR/Cas9 system, sgRNA nucleotides were cloned into pSpCas9(BB)-2 A-GFP (purchased from Addgene, #48138) at BbsI site as described previously [26]. Cell transfection was carried out using Turbofect (Thermo Scientific) following the manufacturer’s protocol. Briefly, DLD1 and HT29 cells were plated into 6-well plates (1 × 10^6^ cells/well) 24 h prior transfection. Next, a mixture containing 2 µg of pSpCas9(BB)-2 A-GFP plasmid constructs and 10 µL of Turbofect in final volume of 300 µl Opti-MEM (Thermo Scientific) growth medium was incubated at room temperature for 20 min and added to the cells. 48 h following cell transfection, GFP positive cells were analyzed and sorted using BD FACS AriaIII™ flow cytometer (BD Biosciences, USA). Single cell colonies were obtained by serial dilution of sorted cells plated in 96 well cell culture plates. Generated monoclonal cells were lysed using Phire Tissue Direct PCR Kit (Thermo Scientific) according to the manufacturer’s instructions. Briefly, cell pellets were lysed in 20 µl of lysis buffer including 0.5 µl DNA Release Additive at 98 °C for 5 min. Genomic PCR was carried out using 0.75 µl of cell lysates and the primers listed in Additional Table 2. PCR amplification was carried out in a SensoQuest Labcycler (Germany). Lastly, genome editing was verified performing T7 endonuclease assays according to manufacturer’s instructions (New England Biolabs). Successfully modified clones were selected for further experiments.

### Immunoblotting

Total protein was extracted from cells 48 h after plating (1 × 10^6^ cells per T25 flask). Cells were lysed in RIPA lysis buffer supplemented with 1x protease inhibitor (Thermo Scientific). Western blot analysis was performed by standard immunoblotting following SDS-polyacrylamide gel electrophoresis using 4% stacking gels and 8% separating gels. Protein extracts were analyzed with the following primary antibodies: *PARP9* (Thermo Scientific, cat. no. 40-4400; 1:1000), *PARP12* (Thermo Scientific, cat. no. PA5-51967; 1:1000), *PARP13* (Thermo Scientific, cat. no. PA5-31650; 1:5000), *PARP14* (Santa Cruz, cat. no. sc-377150; 1:1000), actin (Abcam, cat. no. ab8227; 1:10000). The following secondary antibodies were used at a 1:1000 dilution: Rabbit IgG HRP-conjugated (R&D Systems, cat. no. HAF008) and Mouse IgG HRP-conjugated (R&D Systems, cat. no. HAF018). Visualization of membranes was performed using Intas Chemocam Imager system (Royal Biotech, Germany).

### Cell irradiation

Cells were irradiated at room temperature with 6MV X-rays using Varian clinical linear accelerator (Clinac 600 C/D; Varian Medical Systems Inc., Palo Alto, CA, USA). Two different irradiation regimes were used: a single dose of 6–10 Gy and a fractionated dose delivered daily in 5 fractions, 2 Gy per fraction (5 × 2 Gy). The 10 Gy single dose was administered only in the initial evaluation of PARP gene expression, whereas subsequent experiments facilitated the administration of a single 6 Gy dose, since its biologically effective dose (1 × 6 Gy BED = 18.0) is more comparable to 5 × 2 Gy regimen (BED = 16.7 Gy). The dose rate was ~ 3 Gy/min. In all experiments the same experimental design and separate controls of nonirradiated cells were used for all regimens.

### Clonogenic survival assay

DLD1 and HT29 cells were plated in 6‑well plates for 2D (500 cells/well) or embedded in 96 well plates for 3D (500–1000 cells/well) 24 h prior to irradiation and treated with single dose of 6 Gy or fractionated dose of 2 Gy of ionizing radiation (IR) daily for 5 days. In total, 8 days after irradiation DLD1 and HT29 cell colonies (> 50 cells/colony) were fixed with methanol, stained with crystal violet and counted manually. Clonogenic survival was evaluated as described previously [22]. The mean cell survival fraction from 3 independent experiments was used to represent survival at each irradiation dose.

### Spheroid growth assay

DLD1 and HT29 cell spheroids were plated 48 h prior to irradiation. Control and irradiated spheroids were cultivated on separate plates throughout the duration of experiments (overall 14 days including the plating of MCS). Every two days post irradiation, MCSs were imaged with an inverted optical microscope Eclipse TS100 using digital camera DS-Fi2 (Nikon, Japan). Spheroid size was evaluated using SpheroidSizer 1.0.

### MCS cell survival assay

The viability of DLD1 and HT29 cell speroids was measured using a CellTiter-Glo 3D Cell Viability Assay kit (Promega, USA) following the manufacturer’s instructions. Briefly, 48 h after the final fraction of 5 × 2 Gy irradiation the individual spheroids were transferred into individual wells of a white opaque 96-well plate (Thermo Scientific). CellTiter-Glo 3D reagent was then added to each well and the intensity of chemiluminescence was measured 30 min later using the Variokan LUX multiplate reader (Thermo Scientific). In parallel, non-irradiated MCS were utilized as controls.

### RNA extraction

Cells, cultured in 2D, lr-ECM 2D, lr-ECM 3D, or MCS conditions, were harvested after 2 or 6 days of growth, depending on the experimental design performed. In cases involving cell irradiation (single dose or multifractionated) RNA was extracted 4 h after the final dose delivery. Total RNA was isolated using Quick RNA MiniPrep Kit (Zymo Research) following manufacturer’s instructions. For rectal tissue samples, total RNA was isolated using MirVana miRNA isolation Kit (Thermo Scientific) as per manufacturer’s instructions. The quantity and quality of RNA were evaluated using Nanodrop 2000 (Thermo Scientific). Subsequent RNA integrity assessment, based on the evaluation of 28 S and 18 S ribosomal subunit peaks in electropherogram, was performed using Bioanalyzer 2100 (Agilent, USA).

### Microarray analysis

cRNA sample preparation, labeling and hybridization was performed according to manufacturer’s instructions. Briefly, 100 ng of total RNA was used for cDNA synthesis and amplification using Low Input Quick Amp Gene Expression Labeling Kit (Agilent). Then 825 ng of cRNA labeled with Cy3/Cy5 dyes using Gene Expression Hybridization Kit (Agilent) were hybridized to Human 4 × 44k Oligonucleotide Microarrays (Agilent) using HS 400 hybridization station (Tecan, Switzerland). Microarray slides were scanned using SureScan Microarray scanner (Agilent). Microarray image analysis and data generated were further analyzed using ImaGene ver. 9.0 (BioDiscovery, USA) and GeneSpring GX v11.5 software (Agilent, USA). Loess normalization was performed to adjust microarray data for variation. Gene expression fold change above 1.5 (with *p*-value < 0.05) was defined as differentially expressed between two conditions. Microarray design and data are available at the GEO database (*Accession No. GSE280331*,* GSE280332*).

### Microarray data enrichment analysis

Kyoto Encyclopedia of Genes and Genomes (KEGG) pathway enrichment analysis of gene expression data was performed using the WEB‑based Gene SeT AnaLysis Toolkit, as described previously [27]. P‑values were calculated using the hypergeometric test and adjusted using the Benjamini and Hochberg procedure. Functional KEGG pathway categories associated with ≥ 5 genes were considered as significantly enriched (*p* < 0.05) in differentially expressed genes.

### RT-qPCR

cDNA was synthesized using Revert Aid RT Kit (Thermo Scientific) according to manufacturer’s instructions. A total of 1 µg RNA was used for cDNA synthesis. Quantitative real-time PCR was performed using QuantStudio5 thermal cycler (Applied Biosystems, USA) and Maxima SYBR Green qPCR Master Mix (Thermo Scientific) according to manufacturer’s instructions. The relative changes in gene expression were calculated by ΔΔCt method using *GAPDH* as the housekeeping gene (for cell line experiments) or *GAPDH*, *ACT* and *TBP* geomean (for patients’ tissue samples) for sample normalization [28]. Primer sequences are shown in Additional Table 2.

### Statistical analysis

The statistical analysis was performed using the data analysis software package SPSS 26.0 (IBM) and GraphPad v9.0 (GraphPad Software). The statistical significance of gene expression and surviving fraction differences in cells was calculated using an unpaired t-test. For differences of spheroid growth kinetics evaluation, two-way ANOVA was used. Each data point is displayed as the mean ± standard deviation of at least three independent biological experiments. Normal distribution of data was verified using Shapiro-Wilk test. Differences in PARP expression levels in rectal tissue samples were evaluated using paired t-test. Patients were separated into two groups based on gene mean expression (cut-off value). Samples with less than mean expression level were assigned to the low expression group; samples with above mean value were assigned to the high expression group. A two-sided Chi-square test or Fisher’s exact test were used to analyze the distribution of cases with low or high studied genes level in tumor samples according to the demographic and clinicopathological characteristics of the patients. The overall survival (OS) and progression-free survival (PFS) were evaluated by Kaplan-Meier analysis and log-rank test. Univariate and multivariate Cox regression analysis was performed to detect independent factors significantly determining OS or PFS. Bioinformatic analysis of TCGA data (expression and survival) was performed by GEPIA2 online software [29]. The observations with a p-value of less than 0.05 were considered significant.

## Results

### Ionized radiation affects *PARP* gene expression in colorectal cancer cells in a microenvironment dependent manner

**Fig. 1:**
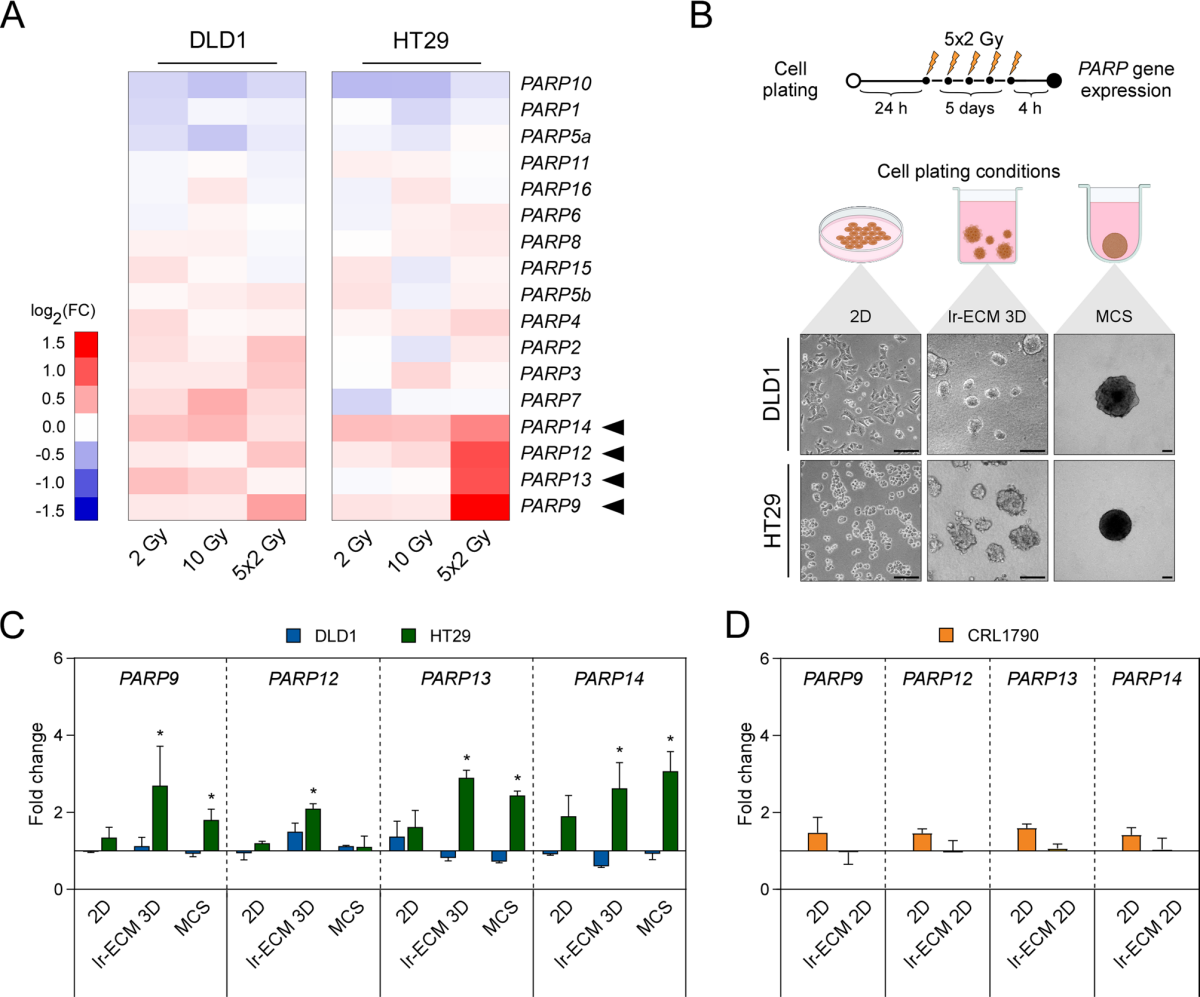
Ionizing radiation induces PARP gene expression in colorectal cancer cells in a microenvironment dependent manner. (**A**) Heatmap analysis depicts the expression pattern of *PARP* family genes in laminin-enriched 3D cell cultures of DLD1 and HT29 cells following multifractionated (5 × 2 Gy) dose ionizing radiation treatment. Unirradiated cells were used as a control. The microarray dataset originates from a previous study (GSE75551). (**B**) Multifractionated irradiation experimental design. 24 h after cell plating, cells were irradiated with a single dose of X-ray (2 Gy) every 24 h for 5 days, resulting in a total dose of 10 Gy. RNA for gene expression analysis was extracted 4 h after cell irradiation. Below are schematics and representative phase-contrast images of CRC cells grown under three different plating conditions: monolayer culture (2D), three-dimensional laminin rich-extracellular matrix culture (lr-ECM 3D), and multicellular spheroid (MCS) system. Scale bars indicate 200 μm. (**C**) Expression of *PARP9*,*12*,*13*,*14* genes was examined using RT-qPCR in colorectal cancer cells (DLD1, HT29) cultivated under 2D or both 3D cell culture conditions after exposure to multifractionated irradiation (5 × 2 Gy). (**D**) Similarly, the expression of *PARP9*,*12*,*13*,*14* was investigated in normal colon cells (CRL1790) grown in a monolayer or lr-ECM coated 2D culture, following the same regimen of irradiation (5 × 2 Gy). Results show means with error bars representing standard deviation (*n* = 3, *p < 0.05, Student’s t-test)

Guided by genome-wide transcriptomics data [22] in colorectal cancer cells exposed to single (2–10 Gy) or multifractionated of a 2 Gy dose of X-ray ionizing irradiation every 24 h for 5 days (Fig. 1A), we selected four PARP genes including *PARP9*, *PARP12*, *PARP13* and *PARP14* and implemented a more detail analysis of selected gene expression levels in non-irradiated cancer and non-cancerous cell lines and cells subjected to multifractionated 5 × 2 Gy irradiation (Fig. 1B). The experiments were performed using three different cell plating conditions: DLD1, HT29, HCT116 and SW48 cells were grown as plastic-attached monolayer (2D), scaffold-attached cells in laminin-rich extracellular matrix (lr-ECM 3D) or scaffold-free multicellular spheroids (MCS) (Fig. 1B, Additional Fig. 1A). For normal colon CRL1790 cells, we selected a monolayer model system with a laminin-rich ECM pre-coated surface (Additional Fig. 1B).

Quantitative PCR analysis revealed that multifractionated irradiation significantly upregulated *PARP9*, *PARP12*, *PARP13* and *PARP14* expression in HT29 cells cultured in both lr-ECM 3D and MCS (FC > 1.5, *p* < 0.05) (Fig. 1C) whereas no significant change in expression was observed in cells grown as a monolayer compared to non-irradiated cells. In contrast, DLD1, HCT116 and SW48 cells did not show such *PARP* expression response to multifractionated irradiation (Additional Fig. 2). Interestingly, irradiated normal colon cells exhibited only a modest effect on *PARP* expression when cultured in 2D but not in lr-ECM (Fig. 1D). Altogether, these preliminary findings suggest that the expression of *PARP9*, *PARP12*, *PARP13* and *PARP14* in irradiated cells is reliant on 3-dimensional cellular microenvironment or cell type, and the *PARP* expression pattern may be specific to cancer cells.

**Fig. 2:**
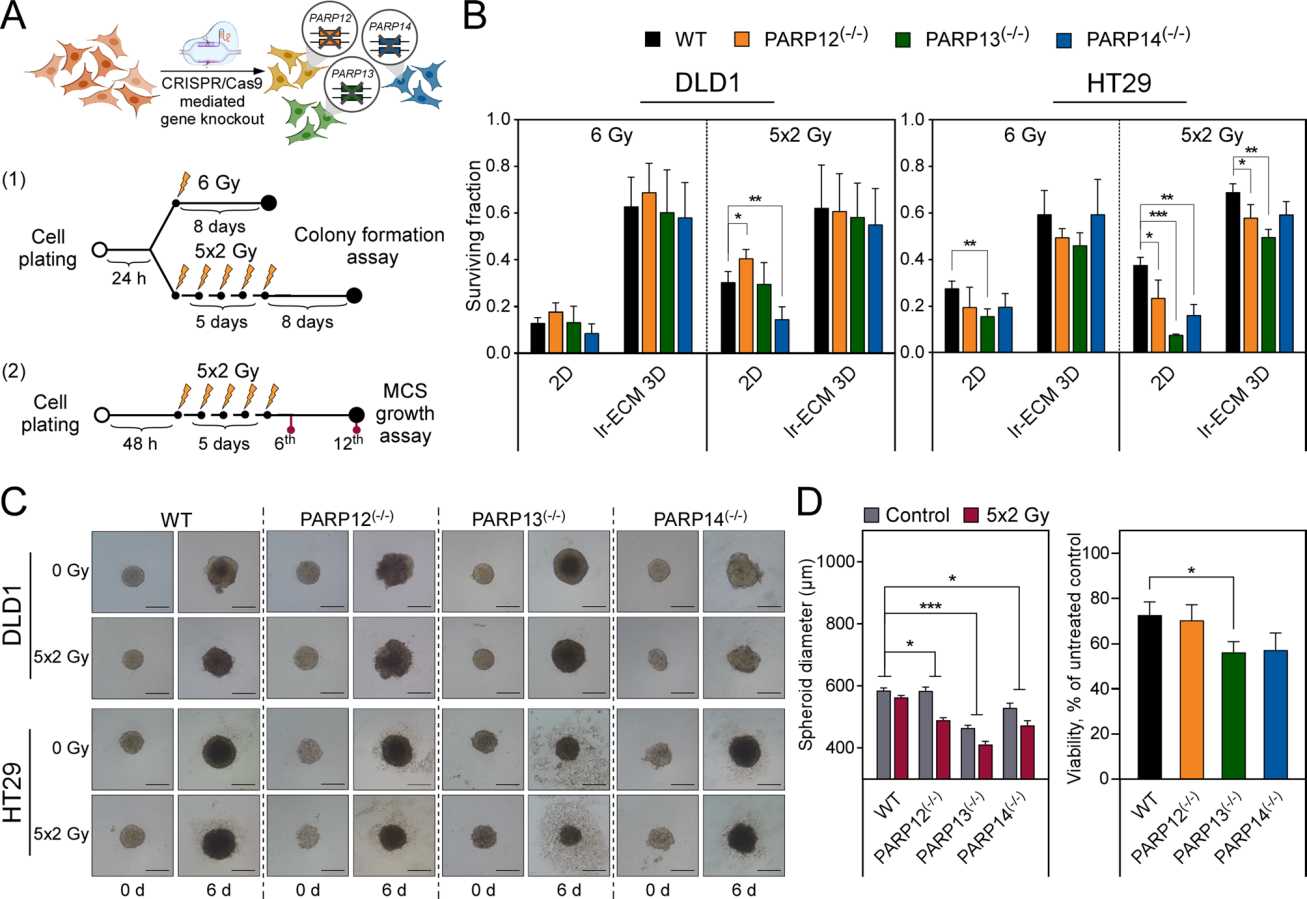
PARPs regulate colorectal cancer cell radiosensitivity in a microenvironment-dependent manner. (**A**) DLD1 and HT29 cell sublines with indicated PARP knockouts were generated using CRISPR/Cas9 genome editing. Single dose and multifractionated irradiation experimental design for colony formation assay (1) and MCS growth assay (2). (**B**) Clonogenic survival of CRC PARP knockout cells after irradiation with a single dose (6 Gy) or multifractionated (5 × 2 Gy) regimens. Wild type cells were used as the control. Results show means with error bars representing standard deviation (*n* = 3, *p < 0.05, **p < 0.01, ***p < 0.001, Student’s t-test). (**C**) Morphology of CRC PARP knockout cells spheroids, (**D**) their growth kinetics and viability following multifractionated irradiation treatment. Representative images show the spheroids grown at the optimal seeding densities between day 0 and 6 of irradiation treatment. Scale bars indicate 200 μm. The growth kinetics are represented by spheroid diameter on the 6th day of irradiation treatment; same time point for MCS viability. Unirradiated spheroids were used as the control. Results show means with error bars representing standard deviation (*n* = 3, *p < 0.05, **p < 0.01, ***p < 0.001, during statistical evaluation, two-way ANOVA for MCS diameter, Student’s t-test for MCS viability)

### *PARP13* is the most prominent modulator of CRC cell sensitivity to multifractionated irradiation

To investigate the role of PARP genes in colorectal cancer cell response to ionizing irradiation, we generated *PARP12*, *PARP13*, and *PARP14* knockout (KO) sublines of DLD1 and HT29 cells using CRISPR/Cas9 gene editing (Additional Fig. 3). The produced PARP KO subclones, along with the WT control lines, were evaluated for cell viability in 2D and lr-ECM 3D cell culture settings after the irradiation with a single (6 Gy) or multifractionated (5 × 2 Gy) dose as shown in Fig. 2A. Colony formation analysis (Fig. 2B, Additional Fig. 4) revealed that DLD1 PARP KO cells were as radiosensitive as wild type cells when treated with a single dose of ionizing radiation in both cell culture conditions, whereas multidose exposure significantly impaired the survival of *PARP12*^(−/−)^, *PARP14*^(−/−)^ cells grown in monolayer, but not in lr-ECM 3D. In contrast, HT29 cells exhibited a distinct profile of PARP-dependency on the response to ionizing irradiation. While only *PARP13* KO dramatically increased the susceptibility of HT29 cells to a single-dose irradiation in 2D, monolayer HT29 *PARP12*^(−/−)^, *PARP13*^(−/−)^ and *PARP14*^(−/−)^ sublines exhibited significantly reduced survival upon exposure to 5 × 2 Gy. Notably, *PARP13* KO had the most dramatic effect, reducing the surviving cell fraction down to 7.4%. Remarkably, a significant level of radiosensitization was retained in HT29 *PARP12*^(−/−)^ and *PARP13*^(−/−)^ within lr-ECM 3D cultures when exposed to multifractionated irradiation, exposing *PARP13* KO cells as more susceptible to radiation than *PARP12* KO cells as well. Overall, these results indicated that *PARP13* KO had the most pronounced effect on HT29 cell radiosensitivity, significantly reducing colony formation in both 2D and lr-ECM 3D growth conditions (*p* < 0.001 and *p* < 0.01, respectively) when using a clinically relevant radiotherapy regimen.

**Fig. 3:**
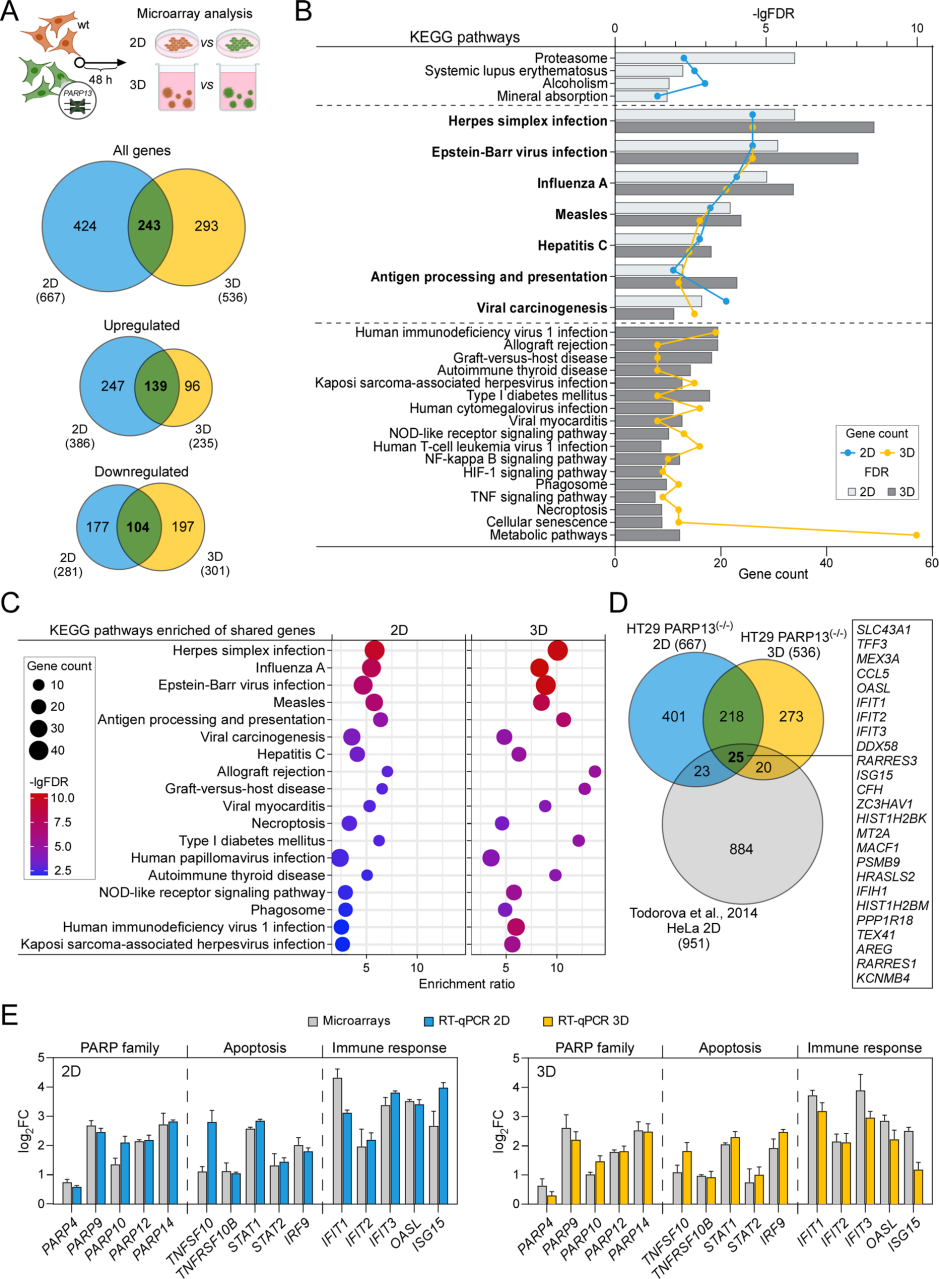
*PARP13* regulates immune gene expression in CRC cells. (**A**) Experimental design of microarray analysis. Total RNA was extracted 48 h after plating HT29 wild type and *PARP13* knockout cells in 2D (blue) or lr-ECM 3D (yellow) culture conditions. Venn diagrams illustrate differentially expressed genes (DEGs) in HT29 *PARP13* knockout cells compared to wild type cells, determined using Agilent array analysis of total mRNA (*n* = 3 independent experiments). (**B**) KEGG pathways significantly enriched in HT29 *PARP13* knockout cells grown under 2D and lr-ECM 3D conditions. Shared pathways commonly enriched in both cell culture conditions are highlighted in bold. The upper X-axis represents -lgFDR values, the lower X-axis indicates the number of genes in each pathway. The Y-axis shows the enriched KEGG pathways. (**C**) KEGG pathway enrichment analysis of overlapped genes between 2D and lr-ECM 3D systems, encompassing both upregulated and downregulated genes. The Y-axis represents significantly enriched KEGG pathways, the X-axis displays the enrichment ratio, and the color of the dots signifies -lgFDR values. The size of the dots corresponds to the number of genes enriched in the respective KEGG pathways. (**D**) Comparative Venn’s diagram analysis showing amount of DEGs in comparison to an independent *PARP13* knockout experiment in HeLa cell line (GSE56667). The box emphasizes genes commonly expressed among all three groups. (**E**) Microarray data validation using RT-qPCR analysis. Bars indicate fold change of representative upregulated genes belonging to three distinct groups: PARP family, apoptosis, and immune response in HT29 *PARP13* knockout cells compared to wild type cells. The results display means with error bars representing standard deviation (*n* = 3, *p* < 0.05, Student’s t-test)

**Fig. 4:**
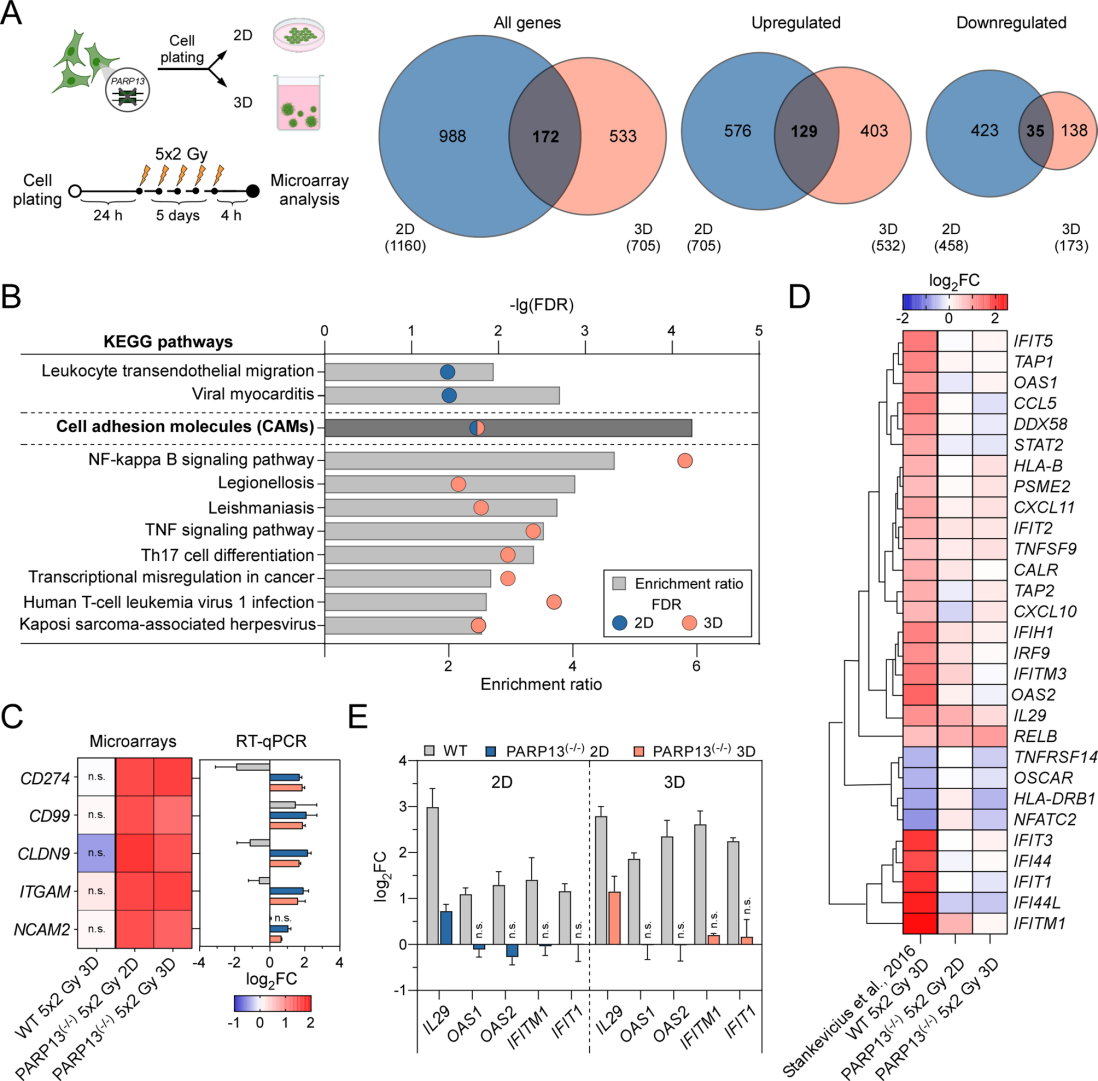
*PARP13* regulates immune gene expression in ionizing radiation treated CRC cells. (**A**) Experimental design of microarray analysis for multifractionated irradiation treated HT29 *PARP13* knockouts in both 2D and lr-ECM enriched 3D conditions. Cells were irradiated with a single dose of X-ray (2 Gy) every 24 h for 5 days, resulting in a total dose of 10 Gy. Total RNA was extracted 4 h post cellular irradiation. Venn diagrams illustrate differentially expressed genes (DEGs) in 2D (blue) and lr-ECM 3D (pink) conditions from global analysis in irradiated HT29 *PARP13* knockouts relative to non-treated *PARP13* knockout cells, determined using Agilent array analysis of total mRNA (*n* = 3 independent experiments). (**B**) Significantly enriched KEGG pathways in irradiated *PARP13* knockout cells grown under 2D and lr-ECM 3D conditions. The upper X-axis represents -lgFDR values, the lower X-axis indicates the enrichment ratio, and the Y-axis shows the enriched KEGG pathways. (**C**) Microarray data validation using RT-qPCR analysis. Results are presented as means with error bars representing standard deviation (*n* = 3, Student’s t-test, n.s.– not significant). (**D**) Clustering heatmap of immune response related gene expression data in irradiated HT29 wild type (GSE75551) and *PARP13* knockout cells under 2D or lr-ECM enriched 3D conditions. (**E**) Confirmation of differential expression of selected four immune response-related genes in HT29 wild type and *PARP13* knockout irradiated cells using RT-qPCR. Results demonstrate means (*n* = 3, Student’s t-test, n.s.– not significant)

To further validate the impact of PARPs on colorectal cancer cell susceptibility to ionizing radiation, we irradiated CRC multicellular spheroids with 5 × 2 Gy and monitored the MCS morphological integrity and growth rate dynamics over a two-week period (Fig. 2A). Surprisingly, the initial spheroid growth analysis revealed that *PARP13* deficiency affects spheroid size even before irradiation, as HT29 *PARP13* KO spheroids were the smallest in diameter among all PARP KO variants compared to wild type cells (Additional Fig. 5). On the other hand, irradiation did not affect DLD1 WT and *PARP13*^(−/−)^ spheroid structural integrity, while *PARP12*^(−/−)^ and *PARP14*^(−/−)^ spheroids started to disintegrate 6 days after treatment initiation. In contrast, all HT29 PARP KO spheroids exhibited visible disintegration at the edge level (Fig. 2C, Additional Fig. 7A). As expected, HT29 *PARP13*^(−/−)^ spheroids were particularly susceptible to disintegration, suggesting a role for *PARP13* in maintaining spheroidal structure via cellular adhesion or cellular survival. Moreover, HT29 *PARP12*^(−/−)^ and *PARP13*^(−/−)^ spheroids experienced a considerable reduction in size following irradiation (Fig. 2D and Additional Fig. 6), supporting the impact of PARPs on colorectal cancer cell sensitivity to IR in the microenvironment dependent manner. Supporting these observations, spheroid viability assays confirmed that HT29 *PARP13* cells exhibited the greatest sensitivity to fractionated dose irradiation, while knockouts of other PARP genes did not significantly alter radiation sensitivity in either HT29 or DLD1 cells (Fig. 2D, Additional Fig. 7B and C). These results, validated by both spheroid size and viability assays, underscore the role of *PARP13* in modulating cellular response to ionizing radiation.

**Fig. 5:**
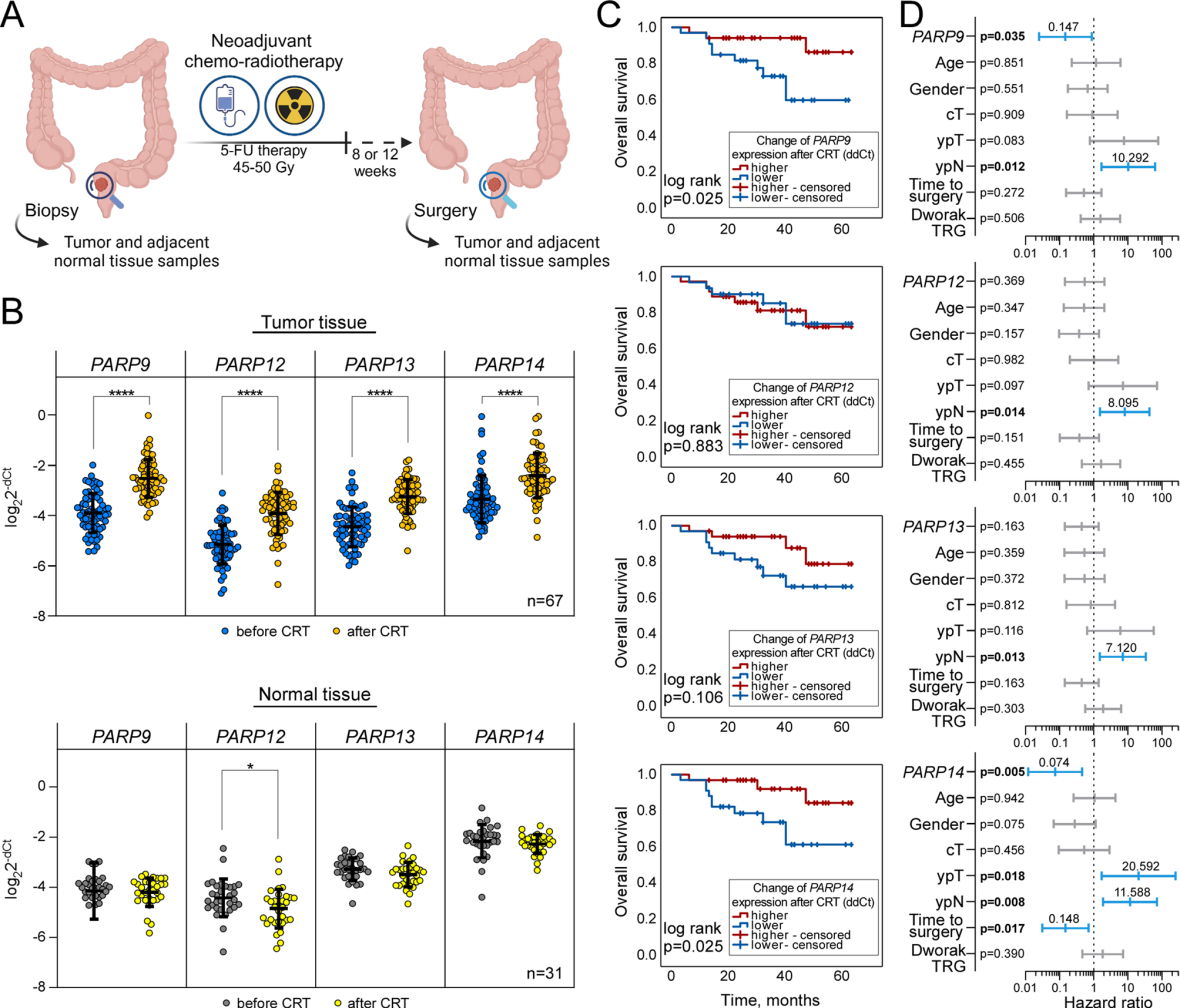
Association of *PARP9*,*12*,*13*,*14* gene expression with rectal cancer clinical outcome following chemoradiotherapy (CRT) treatment. (**A**) Schematic overview of sample collection from rectal cancer patients. Tumor and adjacent normal tissue samples were obtained through biopsies patients with suspected rectal cancer. Following confirmation of diagnosis, patients underwent neoadjuvant chemoradiotherapy involving 5-fluorouracil before surgical resection of tumor. During the tumor resection surgery, additional samples of tumor and adjacent normal tissue were collected from each patient for further analysis. (**B**) qRT-PCR analysis of relative *PARP* gene expression levels before and after CRT in tumor and normal tissue sample groups. The cycle threshold (Ct) values of target genes were normalized to *GAPDH*,* ACT* and *TBP* levels. Lines within boxes indicate relative gene expression mean values, while whiskers denote standard deviation of the relative gene expression values (tumor *n* = 67, normal *n* = 31, Student’s t test, *p < 0.05 and ****p < 0.0001). (**C**) Kaplan-Meier survival curves demonstrating the association between changes in *PARP* expression after CRT and overall survival (OS) in rectal cancer tissue samples (*n* = 67). Patients were stratified into high and low change of expression groups according to the mean value. Curves were compared using the log-rank test, *p* values shown. (**D**) Prognostic performance of *PARP* genes expression changes and clinicopathologic features by multivariate Cox regression analysis. Forest plot illustrates the hazard ratio (vertical bar and number above it), and 95% confidence intervals (whiskers) associated with predictors for rectal cancer patients’ OS in tumor samples (*n* = 67). Significant predictors are highlighted in blue, with displayed *p* values

Additionally, a parallel survival analysis showed that reduced levels of *PARP9* in HT29 cells (Additional Fig. 8A and B) were associated with reduced viability after a single 6 Gy irradiation, while no other changes in radiosensitivity were observed in cells grown in 3D cell models compared to WT cells (Additional Fig. 8C and D).

Overall, our observations underscored the radiosensitizing effect of selected PARPs which was cancer cell type and cellular microenvironment dependent proposing *PARP13* as an essential gene for maintaining HT29 cell survival under the stress of radiation exposure. *PARP13*^(−/−)^ cells exhibited increased sensitivity to both single-dose and multifractionated ionizing radiation regimens, leading to an impaired survival and spheroidal integrity.

### *PARP13* regulates the expression of genes associated with immune response in CRC cells

Having demonstrated that the loss of *PARP13* is associated with increased CRC cell sensitivity to multifractionated irradiation in a microenvironment dependent manner, we went on to the level of whole genome transcriptome to compare the relative abundance of transcripts in HT29 *PARP13*^(−/−)^ cells to wild type cells cultured in 2D and lr-ECM 3D model systems (Fig. 3A). Microarray analysis revealed significant changes in gene expression, with 667 and 536 differentially expressed genes (DEGs) in 2D and lr-ECM 3D, respectively, exposing a lesser deregulation of gene expression in 3D cultures. Conversely, a higher number of genes were downregulated in 3D as well. Further analysis of overlapping 243 DEGs between cells cultured in 2D and 3D showed a slightly higher number of upregulated genes suggesting *PARP13*-related relaxation of specific transcripts.

The latter KEGG analysis showed that overlapping genes were primarily involved in multiple pathways associated with immune response (Fig. 3B, Additional Tables 3 and 4). Notably, these pathways were more significantly enriched in DEGs when cells were cultivated in 3D culture (Fig. 3C, Additional Table 5), suggesting that *PARP13* could regulate cancer cell immune response signaling in a microenvironment dependent manner.

To further assess the specificity of *PARP13* related gene deregulation, we also compared our DEG list with gene expression data set resulted from *PARP13* silencing experiments in HeLa cells [30] (Fig. 3D). Although we identified a set of only 25 DEGs common among three compared gene sets, the profile was strongly associated with immune pathways indicating a significant role for *PARP13* in the regulation of cellular immune responses cancer cell lines of different origins.

To verify the microarray data, we selected genes associated with PARP family (*PARP4*, *PARP9*,* PARP10*, *PARP14*), apoptosis (*TNFSF10*,* TNFRSF10B*,* STAT1*,* STAT2*,* IRF9*), and immune response pathways (*IFIT1*,* IFIT2*,* IFIT3*,* OASL*,* ISG15*). qRT-PCR results largely confirmed the microarray data trends observed in HT29 cells under both 2D and lr-ECM 3D growth conditions (Fig. 3E). Notably, *PARP13* itself emerged as a regulator of other PARP family members expression levels, with the strongest effect on *PARP9* and *PARP14*. Furthermore, HT29 *PARP13*^(−/−)^ cells exhibited induced expression of known proapoptotic genes *TNFSF10*, *TNFRSF10B* and *IRF9* proposing a pro-survival function for *PARP13*. Additionally, the highest upregulation was observed in immune response genes, further highlighting the potential involvement of *PARP13* in immune regulation. While the microarray data showed no clear difference in gene expression levels between 2D and 3D cultures for the selected genes (Additional Fig. 9A), qRT-PCR validation revealed some discrepancies. Notably, the increase in *OASL* and *ISG15* expression, genes related to the immune response, was lower in 3D compared to 2D. In contrast, *PARP13* status did not correlate with expression of PARP family genes in DLD1 cells (Additional Fig. 9B), while the expression of apoptosis and immune pathways-related genes showed a much lower response compared to HT29 cells suggesting that *PARP13* regulatory targets might be cell type dependent.

Taken together, these findings indicate that *PARP13* status in CRC cells correlates with the expression regulation of genes associated with immune response signaling pathways, potentially impacting the PARP family regulation itself. Furthermore, *PARP13*’s role in the regulation of survival genes in CRC cells might be microenvironment dependent.

### Loss of *PARP13* impairs immune response in CRC cells triggered by multifractionated radiation

Given that *PARP13* might impact CRC cell radiosensitivity in a microenvironment dependent manner, we sought to expose core molecular pathways influenced by *PARP13*-dependent regulation in irradiated HT29 *PARP13*^(−/−)^ cells grown in both 2D and lr-ECM 3D cultures (Fig. 4A). The microarray analysis yielded a higher number of differentially expressed genes in 2D cultured cells (1160) compared to 3D (705), of which only 172 DEGs overlapped between both growth conditions, suggesting that cellular response is significantly microenvironment dependent. Notably, KEGG analysis revealed that DEGs resulting from both conditions were primarily enriched in cell adhesion molecules (CAMs) (Fig. 4B, Additional Tables 6 and 7). The following RT-qPCR analysis of selected five CAM genes (*CD274*,* CD99*,* CLDN9*,* ITGAM*,* NCAM2*) confirmed a potential role for CAMs in the response to irradiation with *PARP13* deficiency (Fig. 4C). However, a parallel evaluation of selected gene expression in HT29 WT cells highlighted a key difference: the response to irradiation corresponded to *PARP13* status in cells, but not to cell environment conditions. In contrast, irradiated DLD1 *PARP13*^(−/−)^ cells exhibited a different expression pattern compared to HT29 cells (Additional Fig. 10A).

Furthermore, pathway analysis revealed a significant number of genes enriched in immune response pathways in a microenvironment dependent manner (Fig. 4B). Previously we have identified immune response as the most significantly altered in HT29 WT cells after multifractionated irradiation [22], therefore we next investigated a possible impact of *PARP13* in regulating immune response genes. We compared the gene list from HT29 *PARP13*^(−/−)^ microarray analysis with previous dataset obtained from HT29 wt cells cultured in 3D. Hierarchical heat map cluster analysis (Fig. 4D) revealed a striking difference in the immune related gene expression response to irradiation between HT29 wt and *PARP13*^(−/−)^ cells. Wild type cells exhibited a strong activation of the selected genes whereas *PARP13* KO cells displayed a diminished response with minimal expression changes in both 2D and 3D. These findings suggested that *PARP13* may play a critical role in activating immune response upon ionizing radiation treatment under lr-ECM 3D conditions. To confirm the differential response of immune related genes in HT29 *PARP13*^(−/−)^ cells upon irradiation (Fig. 4E), we next performed RT-qPCR analysis of selected *IL29*,* OAS1*,* OAS2*,* IFITM1*, *IFIT1* genes. Notably, *OAS1*,* OAS2*, *IFITM1* and *IFIT1* did not respond to irradiation in *PARP13*^(−/−)^ cells grown in either 2D or 3D, validating the impact of *PARP13* on selected gene expression in CRC cells. On the other hand, a parallel investigation in irradiated DLD1 *PARP13*^(−/−)^ cells exposed a cell type specific response indicating only a differential pattern of *OAS2*, *IFITM1* and *IFIT1* in cells grown in monolayer (Additional Fig. 10B).

Taken together, the microarray data revealed that *PARP13* deficiency in HT29 cells significantly disrupts the activation of immune response signaling pathways upon multifractionated ionizing irradiation.

### Chemoradiotherapy induces unconventional *PARP* expression in rectal cancer tissue

To evaluate the prognostic value of selected PARP gene expression levels in CRC, we collected paired tumor and adjacent normal tissue samples from rectal cancer patients both before and after their neoadjuvant chemo-radiotherapy (CRT) course (Fig. 5A). The expression levels of *PARP9*, *PARP12*, *PARP13*, and *PARP14* were analyzed in the selected patient cohort using RT-qPCR (Fig. 5B, for fold change plots see Additional Fig. 11). Surprisingly, we found that the expression levels of all investigated PARPs were significantly increased in tumor tissue after CRT (*p* < 0.001 in all cases). To our delight, no statistically significant differences were observed in adjacent normal tissue in response to CRT, except the expression level of *PARP12* was found to be slightly lower than before CRT (*p* < 0.05) signaling a significantly specific tumor cell PARP-related response to the therapy course.

The diagnostic sensitivity and specificity of selected PARPs expression levels before CRT were examined by ROC curve analysis (Additional Fig. 12A) demonstrating that *PARP13* had the most significant prognostic accuracy (AUC = 0.901, *p* < 0.001). In addition, *PARP13* expression was consistently higher in tumor than in adjacent normal tissues in TCGA colon and rectal adenocarcinoma datasets (Additional Fig. 13A). For the statistical analysis we stratified selected *PARP* expression levels (dCt) achieved before CRT and fold of expression after CRT (ddCt) into high and low based on the average value in each group. We then explored the distribution of low and high fold of expression after CRT of *PARP9*, *PARP12*, *PARP13*, and *PARP14* in both tumor and adjacent normal tissue sample groups from rectal cancer patients. To achieve this, we applied Chi-square or Fisher’s exact tests to identify associations between changes in expression and demographic and clinicopathological characteristics (see Additional Tables 8 and 9). Although changes in *PARP12* expression in tumor tissue were significantly associated with lymph node status after CRT, no other statistically significant PARP associations with patient variables were found.

Next, we assessed the prognostic value of *PARP9*, *PARP12*, *PARP13* and *PARP14* expression in rectal cancer tumor tissues using Kaplan-Meier analysis, calculating the time of overall survival (OS) and progression-free survival (PFS). Upon analyzing OS using *PARPs* expression levels before and after CRT (Additional Fig. 14), we found that prior to treatment only higher *PARP9* expression levels are significantly linked to lower survival rates (*p* = 0.014). The analysis of OS in patients, stratified by changes in PARPs expression in tumor tissue induced by CRT (Fig. 5C), revealed a worse survival prognosis when there is a lower change in *PARP9* and *PARP14* expression (*p* = 0.025 in both cases). However, no statistically significant association was found between selected PARP expression and PFS rates in rectal cancer patients (Additional Fig. 12B). Additionally, analysis of TCGA data on association of *PARP13* expression with overall and progression-free survival confirmed that *PARP13* does not hold significant prognostic value for *PARP13* in colorectal cancer (Additional Fig. 13B). Overall, these findings suggest that *PARP9* and *PARP14* expression profiles could be important prognostic factors for survival in rectal cancer patients.

Subsequently, we analyzed rectal cancer prognostic factors for OS using univariate and multivariate Cox regression analyses of tissue samples (Additional Table 10). The univariate Cox regression analysis (Additional Fig. 12C) identified primary tumor and lymph node status after treatment, along with Dworak tumor regression grade as prognostic factors for the OS. The final multivariate Cox regression analysis (Fig. 5D) uncovered that the change of *PARP9* and *PARP14* expression could be significant independent prognostic factors for overall survival (HR: 0.15, 95% CI: 0.02–0.88, *p* = 0.035 and HR: 0.07, 95% CI: 0.01–0.46, *p* = 0.005, respectively).

Given that multifractionated ionizing radiation induces the expression of immune response genes in vitro, we additionally explored whether the *PARPs* are co-expressed with selected *OAS2*,* IFITM1*, and *IFIT1* genes in rectal cancer patients. The expression analysis (Additional Fig. 15A) showed significant downregulation of *IFITM1* in tumor tissue, while *IFIT1* was upregulated in both tumor and normal tissue after CRT. Additionally, we observed a lower expression level of *OAS2* in normal tissue after CRT. Our co-expression analysis indicated a significant and strong positive correlation between the expression of all selected *PARPs*,* OAS2* and *IFIT1* in tumor tissue suggesting a coregulatory mechanism within the tumor microenvironment (Additional Fig. 16). In contrast, such PARP-immune gene co-expression was not evident in normal adjacent tissue samples displaying only a weak positive correlation between *PARP14* and *IFIT1* expression after CRT, suggesting a tumor tissue-specific association. While gene expression analysis revealed changes in tumor tissue, these did not translate into a significant diagnostic potential based on ROC analysis (Additional Fig. 15B). Finally, Kaplan-Meier and Cox regression analysis did not associate changes in the expression of these genes neither with overall nor progression-free survival of rectal cancer patients (Additional Fig. 17, Additional Table 11).

Taken together, this study demonstrates that CRT significantly increases the expression of *PARP9*, *PARP12*, *PARP13* and *PARP14*, specifically in rectal tumor tissue. Notably, high pre-treatment *PARP9* expression and a weaker post-treatment increase in *PARP9* and *PARP14* expression emerged as independent predictors of worse overall survival in rectal cancer patients. Although *PARP13*’s overall role in rectal cancer tissue was less evident, *PARP13* was the most significant tumor tissue discriminator.

## Discussion

Radiotherapy remains a cornerstone of cancer treatment, with advancements in delivery techniques and treatment significantly improving patient outcomes by minimizing toxicity to healthy tissues [31]. However, limitations persist due to intrinsic radiation resistance in some tumors, necessitating the development of more sophisticated strategies. Radiosensitizers hold promise in enhancing the efficacy of radiotherapy, and the DDR system presents a compelling target for biological therapy strategies [5]. ADP-ribosylation, a key protein modification in early DNA damage sensing, has been implicated in radiosensitization. Studies have shown that PARP inhibitors can increase tumor cell sensitivity to irradiation [10, 32, 33]. Building upon this established role of PARPs in DDR, we sought to broaden our understanding of understudied members of the PARP family, with the aim of identifying novel radiosensitizing agents for colorectal cancer treatment. We demonstrate a complex interplay between the expression of less studied PARPs, cellular microenvironment context and radiation response.

In contrast to monolayer, three-dimensional cell cultures more accurately recapitulate the in vivo tumor microenvironment, encompassing intercellular and extracellular interactions as well as gradients of nutrients and metabolites [34]. Moreover, the tumor microenvironment can have a profound effect on radiotherapy response as factors such as stromal cells interactions, ECM stiffness and mechanical forces might affect treatment efficacy [35]. We have previously shown that colorectal cancer cells grown in laminin-rich 3D culture exhibit clinically relevant gene expression profiles for the investigation of theranostic biomarkers in rectal cancer [23, 36]. Moreover, this experimental design incorporating clinically relevant radiation exposure could assist in discovering putative molecular pathways linked to radioresistance through the utilization of 3D cell culture models as well [22]. In the present study, we suggest that PARP family members exhibit diverse and context-dependent roles in the cellular response to irradiation. We observed a notable upregulation of *PARP9*, *PARP12*, *PARP13* and *PARP14* across three-dimensional cell culture models, including lr-ECM 3D and multicellular spheroids, following clinically relevant multifractionated irradiation. Intriguingly, the observed PARP expression pattern might be unique to cancer cells, as no corresponding expression changes were found in normal cells following irradiation suggesting cancer-specific therapeutic adjuvant intervention (Fig. 1). Together these findings indicate that the radiosensitizing potential of unconventional PARPs should be tested using 3D cell cultures, which may provide a physiologically relevant platform for identification of novel therapeutic strategies.

Previous studies have consistently demonstrated that cells grown in 3D cultures exhibit increased radioresistance compared to 2D cultures [37–39]. Therefore, given the advantages of 3D cell culture in mimicking the in vivo tumor microenvironment, the applications of such cell models could profoundly implicate the development of innovative and effective radiotherapy strategies [40]. Our present findings highlight the radiosensitizing potential of understudied PARPs, with their effect dependent on both cancer cell type and cellular microenvironment (Fig. 2). PARP KO in DLD1 cells resulted in an elevated radiosensitivity cultivating them only in monolayer, while HT29 PARP KO cells remained susceptible to IR in 3D models. *PARP13* emerged as the most critical gene for HT29 cell survival following radiation exposure including 3D cell culture settings. We discovered that HT29 cells lacking *PARP13* display increased sensitivity to both single and multi-fractionated radiation regimens, resulting in impaired survival and a loss of spheroid integrity. Previous findings indicated that *PARP13* knockdown significantly decreased HeLa cell viability via destabilization of *TRAILR4* transcript, thereby sensitizing cells to apoptosis [30, 41], suggesting that the sensitivity to irradiation could be intrinsically related to the dysregulation of cell survival genes due to *PARP13* knockout. In line with these observations, we showed that *PARP13* deficiency resulted in the upregulation of TRAIL and TRAIL-R2/DR5 encoding genes, *TNFSF10* and *TNFRSF10B*, respectively, which could drive the intrinsic susceptibility of cancer cells to apoptosis [42]. Alternatively, radiosensitization could be acquired due to disrupted regulation of IR-induced DDR response genes in *PARP13* KO cells. However, the publicly available data on how *PARP13* could impact cell survival in response to radiation is absent.

*PARP13* is an RNA-binding protein that is primarily involved in the activation of cell antiviral immune response [43]. During viral infection, *PARP13* destabilizes target RNA by direct binding and the recruitment of exosome factors to initiate their decay [44]. In addition, the translation of specific targets could be also repressed by binding of *PARP13* to the eIF4A translation-initiation factor [45]. Regarding these findings, we subsequently revealed that *PARP13* KO in HT29 cells resulted in a strong deregulation of RNA levels of genes involved in viral immune response pathways (Fig. 3**)**, demonstrating that *PARP13* could regulate host mRNA stability in CRC cells. This observation is in agreement with a recent report indicating that *PARP13* could keep the immune response primed for stress stimulus in cell basal state [43]. Moreover, our findings show that *PARP13* might influence the regulation of the PARP family itself. Knocking out *PARP13* led to overexpression of *PARP9*,* PARP10*, *PARP12* and *PARP14*. Despite *PARP13* being catalytically inactive, it could be MARylated by *PARP14*, indicating the interplay among PARP family proteins [46]. Meanwhile, *PARP14* activity is directly regulated by *PARP9*/DT3XL, enabling hydrolytic activity of *PARP9* and DT3XL interaction with *PARP14* [47]. Therefore, our findings illustrate a significant interaction between the respective PARPs existing at RNA levels as well.

In cancer cells, irradiation can promote the formation of micronuclei, whose nuclear envelope is prone to rupture, releasing DNA into cytosol. The exposed cytosolic DNA subsequently triggers IFN signaling cascade leading to the induced transcription of interferon-stimulated genes (ISGs) [48, 49]. Previously, we have showed that fractionated irradiation induced a significant overexpression of immune response genes including ISGs [22]. To our surprise, a subsequent microarray analysis revealed that *PARP13* deficiency in HT29 cells significantly attenuated the activation of immune response pathways (Fig. 4), pointing at *PARP13* as a key regulator of IFN pathway response to IR. Our findings show that IFITM1 and *IFIT1* are among strongly induced in HT29 wild type cells but not in *PARP13* knockout cells. Notably, both IFITM1 and *IFIT1* are part of the interferon-related DNA damage resistance signature (IRDS) genes, which has been linked to the development of resistance to radiation therapy, chemotherapy, and immune checkpoint blockades in various cancers, and promotion tumor growth, metastasis and immune evasion [50, 51]. *IFIT1* is frequently upregulated in DNA damage-resistant cancer cell sublines, including those resistant to radiation or chemotherapy [52] and residual tumors following treatment, such as in estrogen receptor–negative breast cancer following chemotherapy [53]. Similarly, IFITM1 expression is regulated by interferon-alpha/NF-κB signaling, and its suppression has been shown to reduce tumor growth and invasion in triple-negative breast cancer [54]. Together, these findings suggest that both *IFIT1* and IFITM1 contribute to therapy resistance and tumor progression, likely through modulation of interferon and growth factor signaling pathways and may represent potential targets for therapeutic intervention in cancers characterized by high *PARP13* and ISG expression. Moreover, *PARP13* levels are found to be elevated upon IFN stimulation both in vitro and in vivo [55, 56], demonstrating its reciprocal regulation. The activation of ISGs can either stimulate intrinsic cancer cell DDR response to IR via STAT1 pathway [52, 57] or foster tumor immune cell evasion [58, 59] resulting in immunosuppressive tumor microenvironment. As a result, the elevated levels of ISGs are frequently associated with radioresistance in cancer cells [58, 60], proposing that *PARP13* could maintain cell survival by regulating RNA stability of ISGs. Interestingly, we also detected an upregulated expression of *CD274*, encoding PD-L1, in irradiated *PARP13* KO cells, suggesting a potential immunotherapeutic window for tumors with lower *PARP13* expression.

Finally, we demonstrated that chemoradiotherapy significantly upregulates the expression of *PARP9*, *PARP12*, *PARP13* and *PARP14* exclusively within rectal tumor tissues (Fig. 5). This finding, observed solely in cancerous tissues, aligns with our in vitro cell culture results, suggesting a strong clinical relevance. Given these observations, prospective of these genes as therapy targets or therapy response biomarkers could be promising direction for future studies as identification of patients who are more likely to benefit from treatment could lead to more personalized therapy approaches and improved outcomes. Regarding *PARP13*, it emerged as the most significant discriminator between tumor and healthy tissues demonstrating a specific *PARP13* role in cancerous cells. However, our data suggest that *PARP13* contributes to tumor response to radiotherapy through indirect mechanisms, such as the regulation of other PARPs and immune response pathways, rather than serving as a standalone clinical biomarker. Surprisingly, our findings highlighted the potential clinical significance of *PARP9* and *PARP14* in rectal cancer. The high pre-treatment *PARP9* levels and a blunted post-treatment increase in both *PARP9* and *PARP14* expression emerged as independent predictors of poorer overall survival in rectal cancer patients. The observed irradiation induced cross-regulation between *PARP13* and *PARP9* or *PARP14* is particularly noteworthy, given that high pre-treatment *PARP9* expression, and lower changes of both *PARP9* and *PARP14* expression post-CRT correlates with poorer survival outcomes. This indicates that *PARP13* may indirectly modulate treatment response by regulating *PARP9* and *PARP14* related pathways, emphasizing the necessity for further investigation of PARPs in colorectal cancer. Our observations are supported by previous studies which demonstrated an elevated expression of *PARP9* and *PARP14* in many malignancies [61–66] whereas high levels of *PARP14* are associated with poor prognosis in patients [67]. As a result, several *PARP14* inhibitors are being recently utilized in preclinical development [68, 69] suggesting their potential adjuvant applicability in radiotherapy.

### Study limitations

We recognize that the absence of direct protein-level validation in this study limits our ability to precisely map the downstream pathways and their contributions to irradiation response in colorectal cancer cells. Future studies should combine proteomic analyses with detailed functional assays in both wild-type and *PARP13* KO HT29 to fully elucidate the role of *PARP13* in modulating gene regulation, cell survival and therapy resistance in colorectal cancer models.

## Conclusion

To summarize, we demonstrate that interferon-associated PARPs are significantly involved in CRC response to radiotherapy in preclinical and clinical settings. *PARP13* emerged as the most crucial for CRC sensitivity to IR treatment in vitro, serving as a keystone regulating ISG immune response in both nonirradiated or IR treated cells, and is the most significant discriminator between tumor and healthy tissues. Simultaneously, albeit less evident in cell culture, elevated coexpression levels of *PARP9* and *PARP14* were associated with poor patient outcomes after chemoradiotherapy. Taken together, our results propose that a collective focus on the relationship between *PARP9/13/14* axis proteins may promote synergistic therapeutic and theranostic outcomes for radiotherapy development.

## Electronic supplementary material

Below is the link to the electronic supplementary material.

Supplementary Material 1

Supplementary Material 2

## Data Availability

The microarray datasets supporting the conclusions of this article are available in the GEO repository, GSE280331 and GSE280332.

## Abbreviations

CRC: Colorectal cancer
CRT: Chemoradiotherapy
IR: Ionizing radiation
DDR: DNA damage response
SSB: Single stranded break
DSB: Double stranded break
ECM: Extracellular matrix
lr-ECM 3D: Laminin rich extracellular matrix 3D cell culture
MCS: Multicellular spheroid cell culture
DEG: Differentially expressed gene
KO: Knockout
PARP: Poly(ADP-ribose) polymerase
ISG: Interferon stimulated genes
OS: Overall survival
PFS: Progression-free survival
