# Supplementary material for: PARP9-PARP13-PARP14 axis tunes colorectal cancer response to radiotherapy
Source: J Exp Clin Cancer Res. 2025 Jul 11;44:199. doi: 10.1186/s13046-025-03439-y (PMC12247367; doi:10.1186/s13046-025-03439-y)
Supplement: Supplementary file 1 — Supplementary Material 1 [file 13046_2025_3439_MOESM1_ESM.docx]

Additional table 1. List of sgRNA coding oligonucleotides used to knockout PARP genes.

| **Target gene** | **Top oligonucleotide sequence** | **Bottom oligonucleotide sequence** |
| --- | --- | --- |
| *PARP9* | CACCGCTGAAACAAGAGTGAGAGCG | AAACCGCTCTCACTCTTGTTTCAGC |
| *PARP12* | CACCGTGAAAGGCACAAGAGCCGTG | AAACCACGGCTCTTGTGCCTTTCAC |
| *PARP13* | CACCGAAAATCCTGTGCGCCCACGG | AAACCCGTGGGCGCACAGGATTTTC |
| *PARP14* | CACCGTTGGTGTTCAAGTTCTTCGG | AAACCCGAAGAACTTGAACACCAAC |

Additional table 2. List of primer sequences used in RT-qPCR

| **Gene** | **Forward sequence** | **Reverse sequence** |
| --- | --- | --- |
| *PARP9* | GGCCTCGGTGGATGGAATG | GCAAACTAACCCGGATAGTCTCT |
| *PARP12* | GCTTGACAACCGAACACAACC | GGCATAGCTCATTATAGCTCAGG |
| *PARP13* | TCACGAACTCTCTGGACTGAA | ACTTTTGCATATCTCGGGCATAA |
| *PARP14* | TGTTAGTGGAGAACATAAGTGGC | TGAATGGTGCTTGGTACAATCAT |
| *GAPDH* | AGCCACATCGCTCAGACAC | GCCCAATACGACCAAATC |
| *TNFSF10* | TGGCTGTAACTTACGTGTACTT | TCATACTCTCTTCGTCATTGGG |
| *TNFRSF10B* | GCCCCACAACAAAAGAGGTC | AGGTCATTCCAGTGAGTGCTA |
| *STAT1* | ATCAGGCTCAGTCGGGGAATA | TGGTCTCGTGTTCTCTGTTCT |
| *STAT2* | CCAGCTTTACTCGCACAGC | AGCCTTGGAATCATCACTCCC |
| *IRF9* | GCCCTACAAGGTGTATCAGTTG | TGCTGTCGCTTTGATGGTACT |
| *IFIT1* | TCATCAGGTCAAGGATAGTCTG | GGTGTTTCACATAGGCTAGTAG |
| *IFIT2* | AAGCACCTCAAAGGGCAAAAC | TCGGCCCATGTGATAGTAGAC |
| *IFIT3* | TCAGAAGTCTAGTCACTTGGGG | ACACCTTCGCCCTTTCATTTC |
| *OASL* | CTGATGCAGGAACTGTATAGCAC | CACAGCGTCTAGCACCTCTT |
| *ISG15* | CGCAGATCACCCAGAAGATCG | TTCGTCGCATTTGTCCACCA |
| *CXCR4* | ACTACACCGAGGAAATGGGCT | CCCACAATGCCAGTTAAGAAGA |
| *BIRC3* | AAGCTACCTCTCAGCCTACTTT | CCACTGTTTTCTGTACCCGGA |
| *FGFR3* | TGCGTCGTGGAGAACAAGTTT | GCACGGTAACGTAGGGTGTG |
| *LGALS1* | TCGCCAGCAACCTGAATCTC | GCACGAAGCTCTTAGCGTCA |
| *RELT* | GTTCCATGTCAACCATGTTCCT | AGGCAGAAGACAGGGACGAT |
| *TNS4* | AGGACACCAGAACTCCGTTCA | TCTCGGGTGATGTTTGGCTTA |
| *CXCL1* | GCGCCCAAACCGAAGTCATA | ATGGGGGATGCAGGATTGAG |
| *CD274* | TGGCATTTGCTGAACGCATTT | TGCAGCCAGGTCTAATTGTTTT |
| *CD99* | AACCCACCCAAACCGATGC | TGAAAAGCTACCGGAGGAACTA |
| *CLDN9* | CGGCTGCACTGCTTATGCT | GAGGGGATGGAGTAGCCCA |
| *ITGAM* | GCCTTGACCTTATGTCATGGG | CCTGTGCTGTAGTCGCACT |
| *NCAM2* | GGGGTTGCTTGTCAGTAGC | TTCAGGTTCACCAATCGCTGT |
| *IL29* | GGACGCCTTGGAAGAGTCAC | AGCTGGGAGAGGATGTGGT |
| *OAS1* | AGCTTCGTACTGAGTTCGCTC | CCAGTCAACTGACCCAGGG |
| *OAS2* | ACGTGACATCCTCGATAAAACTG | GAACCCATCAAGGGACTTCTG |
| *IFITM1* | CCAAGGTCCACCGTGATTAAC | ACCAGTTCAAGAAGAGGGTGTT |

Additional table 3. Full list of all KEGG Pathways enriched in differently expressed genes in HT29 PARP13 KO versus HT29 wt cells under 2D cell culture conditions.

| **Category** | **Number of genes** | **FDR** | **Genes** |
| --- | --- | --- | --- |
| Herpes simplex infection  (hsa05168) | 26 | 1.10E-06 | *SRPK1, SRSF9, CCL5, DDX58, EIF2AK1, EIF2AK2, GTF2I, HLA-A, HLA-B, HLA-C, HLA-DOB, HLA-E, HLA-F, IFIH1, IFIT1, IRF9, MYD88, NFKBIA, OAS2, OAS3, PML, SP100, STAT1, STAT2, TAP1, TAP2* |
| Proteasome  (hsa03050) | 13 | 1.10E-06 | *PSMA3, PSMA7, PSMB6, PSMC2, PSMC3, PSMD12, PSMA1, PSMA6, PSMB10, PSMB7, PSMB8, PSMB9, PSME2* |
| Epstein-Barr virus infection  (hsa05169) | 26 | 4.00E-06 | *AKT1, CD44, PSMC2, PSMC3, PSMD12, B2M, CCND3, DDX58, EIF2AK2, HLA-A, HLA-B, HLA-C, HLA-DOB, HLA-E, HLA-F, IRF9, ISG15, LYN, MYD88, NFKBIA, OAS2, OAS3, STAT1, STAT2, TAP1, TAP2* |
| Influenza A  (hsa05164) | 23 | 9.24E-06 | *AKT1, TMPRSS2, ADAR, CCL5, DDX58, EIF2AK1, EIF2AK2, HLA-DOB, HSPA8, IFIH1, IRF9, MX1, MYD88, NFKBIA, OAS2, OAS3, PML, RSAD2, STAT1, STAT2, TNFRSF10B, TNFSF10, TRIM25* |
| Measles  (hsa05162) | 18 | 1.51E-04 | *AKT1, ADAR, CCND3, DDX58, EIF2AK1, EIF2AK2, HSPA8, IFIH1, IRF9, MX1, MYD88, NFKBIA, OAS2, OAS3, STAT1, STAT2, TNFRSF10B, TNFSF10* |
| Viral carcinogenesis  (hsa05203) | 21 | 1.32E-03 | *CREB3L1, HDAC3, HIST1H4E, HIST1H4J, KAT2A, CCND3, EIF2AK2, HIST1H2BK, HIST1H2BL, HIST1H2BM, HLA-A, HLA-B, HLA-C, HLA-E, HLA-F, IRF9, LYN, NFKBIA, PMAIP1, PXN, SP100* |
| Hepatitis C  (hsa05160) | 16 | 1.63E-03 | *AKT1, CD81, CLDN2, CLDN7, CLDN4, DDX58, EIF2AK1, EIF2AK2, EIF3E, IFIT1, IRF9, NFKBIA, OAS2, OAS3, STAT1, STAT2* |
| Antigen processing and presentation  (hsa04612) | 11 | 5.56E-03 | *B2M, HLA-A, HLA-B, HLA-C, HLA-DOB, HLA-E, HLA-F, HSPA8, PSME2, TAP1, TAP2* |
| Systemic lupus erythematosus  (hsa05322) | 15 | 5.56E-03 | *HIST1H2AJ, HIST1H3A, HIST1H3G, HIST1H4E, HIST1H4J, H2AFJ, H2AFY, HIST1H2BK, HIST1H2BL, HIST1H2BM, HIST1H3C, HIST2H2AA4, HIST2H3A, HIST2H3D, HLA-DOB* |
| Alcoholism  (hsa05034) | 17 | 1.59E-02 | *CREB3L1, GNG12, HDAC3, HIST1H2AJ, HIST1H3A, HIST1H3G, HIST1H4E, HIST1H4J, H2AFJ, H2AFY, HIST1H2BK, HIST1H2BL, HIST1H2BM, HIST1H3C, HIST2H2AA4, HIST2H3A, HIST2H3D* |
| Mineral absorption  (hsa04978) | 8 | 1.84E-02 | *SLC40A1, ATP1A1, MT1A, MT1B, MT1E, MT1H, MT1X, MT2A* |

Additional table 4. Full list of all KEGG Pathways enriched in differently expressed genes in HT29 PARP13 KO versus HT29 wt cells under lr-ECM 3D cell culture conditions.

| **Category** | **Number of genes** | **FDR** | **Genes** |
| --- | --- | --- | --- |
| Herpes simplex infection  (hsa05168) | 26 | 2.61E-09 | *HLA-DMB, NFKBIA, MYD88, TICAM1, IL15, PML, SP100, FAS, HLA-A, TAP2, EIF2AK2, OAS3, CCL5, OAS1, IRF9, HLA-F, HLA-E, STAT1, DDX58, HLA-G, OAS2, HLA-C, TAP1, IFIH1, HLA-B, IFIT1* |
| Epstein-Barr virus infection  (hsa05169) | 26 | 8.86E-09 | *PIK3R2, HLA-DMB, GADD45B, CD58, NFKBIA, MYD88, CALR, FAS, LYN, HLA-A, TAP2, EIF2AK2, OAS3, OAS1, IRF9, HLA-F, HLA-E, STAT1, DDX58, HLA-G, B2M, ISG15, OAS2, HLA-C, TAP1, HLA-B* |
| Influenza A  (hsa05164) | 21 | 1.22E-06 | *PIK3R2, HLA-DMB, NFKBIA, MYD88, TICAM1, PML, FAS, TNFRSF10B, TNFSF10, ADAR, EIF2AK2, OAS3, CCL5, OAS1, IRF9, STAT1, DDX58, RSAD2, OAS2, IFIH1, MX1* |
| Measles  (hsa05162) | 16 | 6.61E-05 | *PIK3R2, NFKBIA, MYD88, FAS, TNFRSF10B, TNFSF10, ADAR, EIF2AK2, OAS3, OAS1, IRF9, STAT1, DDX58, OAS2, IFIH1, MX1* |
| Antigen processing and presentation  (hsa04612) | 12 | 8.99E-05 | *HLA-DMB, CALR, HLA-A, TAP2, PSME2, HLA-F, HLA-E, HLA-G, B2M, HLA-C, TAP1, HLA-B* |
| Human immunodeficiency virus 1 infection  (hsa05170) | 19 | 3.90E-04 | *PIK3R2, CXCR4, APOBEC3B, NFKBIA, MYD88, CALR, PAK6, FAS, HLA-A, TAP2, APOBEC3C, BST2, HLA-F, HLA-E, HLA-G, B2M, HLA-C, TAP1, HLA-B* |
| Allograft rejection  (hsa05330) | 8 | 3.90E-04 | *HLA-DMB, FAS, HLA-A, HLA-F, HLA-E, HLA-G, HLA-C, HLA-B* |
| Graft-versus-host disease  (hsa05332) | 8 | 6.20E-04 | *HLA-DMB, FAS, HLA-A, HLA-F, HLA-E, HLA-G, HLA-C, HLA-B* |
| Hepatitis C  (hsa05160) | 14 | 6.41E-04 | *PIK3R2, CD81, CLDN2, EIF3E, NFKBIA, TICAM1, EIF2AK2, OAS3, OAS1, IRF9, STAT1, DDX58, OAS2, IFIT1* |
| Type I diabetes mellitus  (hsa04940) | 8 | 7.17E-04 | *HLA-DMB, FAS, HLA-A, HLA-F, HLA-E, HLA-G, HLA-C, HLA-B* |
| Autoimmune thyroid disease  (hsa05320) | 8 | 3.12E-03 | *HLA-DMB, FAS, HLA-A, HLA-F, HLA-E, HLA-G, HLA-C, HLA-B* |
| Viral myocarditis  (hsa05416) | 8 | 5.88E-03 | *HLA-DMB, HLA-A, CD55, HLA-F, HLA-E, HLA-G, HLA-C, HLA-B* |
| Kaposi sarcoma-associated herpesvirus infection  (hsa05167) | 15 | 5.88E-03 | *PIK3R2, NFKBIA, TICAM1, CXCL1, FAS, LYN, HLA-A, EIF2AK2, IRF9, HLA-F, HLA-E, STAT1, HLA-G, HLA-C, HLA-B* |
| Metabolic pathways  (hsa01100) | 57 | 7.04E-03 | *PANK1, MVK, CHPT1, NME7, MAN1A1, PANK2, B3GALT6, LDHA, POMGNT1, BCAT2, IDH2, RPIA, COX5A, ST3GAL2, LPIN3, ATIC, AGMAT, GCLM, NADSYN1, ACSS2, GAL3ST1, UGDH, ALG3, HMGCS2, PYGB, LIPT2, QARS, ACO1, MGLL, SGSH, ALDOA, ACAT2, ASNS, ALDH2, ODC1, SMPD3, CRLS1, PAPSS2, HSD17B2, GDA, HGD, AKR1B10, NAT1, B4GALT5, POLR2E, ALOX5, GANC, GGCT, SAT1, GLUD1, DEGS1, A4GALT, LAMA3, LPCAT2, ADA, CMPK2, BLVRA* |
| NF-kappa B signaling pathway  (hsa04064) | 10 | 7.07E-03 | *CARD10, GADD45B, NFKBIA, MYD88, TICAM1, LYN, CD14, PLAU, BIRC3, DDX58* |
| Viral carcinogenesis  (hsa05203) | 15 | 1.10E-02 | *PIK3R2, CREB3L1, NFKBIA, HIST1H2BM, SP100, LYN, HIST1H2BK, HLA-A, EIF2AK2, IRF9, HLA-F, HLA-E, HLA-G, HLA-C, HLA-B* |
| Human cytomegalovirus infection  (hsa05163) | 16 | 1.16E-02 | *PIK3R2, CREB3L1, CXCR4, NFKBIA, CALR, FAS, HLA-A, TAP2, CCL5, HLA-F, HLA-E, HLA-G, B2M, HLA-C, TAP1, HLA-B* |
| NOD-like receptor signaling pathway  (hsa04621) | 13 | 1.63E-02 | *PANX1, NFKBIA, MYD88, TICAM1, CXCL1, MFN2, BIRC3, OAS3, CCL5, OAS1, IRF9, STAT1, OAS2* |
| Phagosome  (hsa04145) | 12 | 0.019388 | *TFRC, HLA-DMB, CALR, HLA-A, CD14, TAP2, HLA-F, HLA-E, HLA-G, HLA-C, TAP1, HLA-B* |
| Cellular senescence  (hsa04218) | 12 | 0.027394 | *PIK3R2, SLC25A5, CAPN2, GADD45B, RRAS, HLA-A, HLA-F, HLA-E, MRAS, HLA-G, HLA-C, HLA-B* |
| HIF-1 signaling pathway  (hsa04066) | 9 | 0.027895 | *LDHA, PIK3R2, PFKFB3, TFRC, PDK1, ALDOA, INSR, EDN1, SLC2A1* |
| Necroptosis  (hsa04217) | 12 | 0.027895 | *SLC25A5, PYGB, CAPN2, TICAM1, GLUD1, FAS, TNFRSF10B, TNFSF10, EIF2AK2, BIRC3, IRF9, STAT1* |
| Human T-cell leukemia virus 1 infection  (hsa05166) | 16 | 0.029421 | *CDC26, PIK3R2, SLC25A5, HLA-DMB, NFKBIA, CALR, IL15, RRAS, SLC2A1, HLA-A, HLA-F, HLA-E, MRAS, HLA-G, HLA-C, HLA-B* |
| TNF signaling pathway  (hsa04668) | 9 | 0.04592 | *PIK3R2, CREB3L1, EDN1, NFKBIA, IL15, CXCL1, FAS, BIRC3, CCL5* |

Additional table 5. Full list of shared KEGG Pathways enriched in upregulated genes in HT29 PARP13 KO versus HT29 wt cells under 2D and lr-ECM 3D cell culture conditions.

| **2D** | | |  | **3D** | | | |  |
| --- | --- | --- | --- | --- | --- | --- | --- | --- |
| **Genes** | **Number of genes** | **FDR** | **Category** | | **FDR** | **Number of genes** | **Genes** | |
| *CCL5, DDX58, EIF2AK1, EIF2AK2, GTF2I, HLA-A, HLA-B, HLA-C, HLA-DOB, HLA-E, HLA-F, IFIH1, IFIT1, IRF9, MYD88, NFKBIA, OAS2, OAS3, PML, SP100, STAT1, STAT2, TAP1, TAP2* | 24 | 6.21E-10 | Herpes simplex infection  (hsa05168) | | 0 | 25 | *NFKBIA, MYD88, TICAM1, IL15, PML, SP100, FAS, HLA-A, TAP2, EIF2AK2, OAS3, CCL5, OAS1, IRF9, HLA-F, HLA-E, STAT1, DDX58, HLA-G, OAS2, HLA-C, TAP1, IFIH1, HLA-B, IFIT1* | |
| *ADAR, CCL5, DDX58, EIF2AK1, EIF2AK2, HLA-DOB, HSPA8, IFIH1, IRF9, MX1, MYD88, NFKBIA, OAS2, OAS3, PML, RSAD2, STAT1, STAT2, TNFRSF10B, TNFSF10, TRIM25* | 21 | 2.42E-08 | Influenza A  (hsa05164) | | 7.26E-11 | 19 | *NFKBIA, MYD88, TICAM1, PML, FAS, TNFRSF10B, TNFSF10, ADAR, EIF2AK2, OAS3, CCL5, OAS1, IRF9, STAT1, DDX58, RSAD2, OAS2, IFIH1, MX1* | |
| *B2M, CCND3, DDX58, EIF2AK2, HLA-A, HLA-B, HLA-C, HLA-DOB, HLA-E, HLA-F, IRF9, ISG15, LYN, MYD88, NFKBIA, OAS2, OAS3, STAT1, STAT2, TAP1, TAP2* | 21 | 3.29E-07 | Epstein-Barr virus infection  (hsa05169) | | 0 | 24 | *GADD45B, CD58, NFKBIA, MYD88, CALR, FAS, LYN, HLA-A, TAP2, EIF2AK2, OAS3, OAS1, IRF9, HLA-F, HLA-E, STAT1, DDX58, HLA-G, B2M, ISG15, OAS2, HLA-C, TAP1, HLA-B* | |
| *ADAR, CCND3, DDX58, EIF2AK1, EIF2AK2, HSPA8, IFIH1, IRF9, MX1, MYD88, NFKBIA, OAS2, OAS3, STAT1, STAT2, TNFRSF10B, TNFSF10* | 17 | 3.66E-07 | Measles  (hsa05162) | | 1.33E-08 | 15 | *NFKBIA, MYD88, FAS, TNFRSF10B, TNFSF10, ADAR, EIF2AK2, OAS3, OAS1, IRF9, STAT1, DDX58, OAS2, IFIH1, MX1* | |
| *B2M, HLA-A, HLA-B, HLA-C, HLA-DOB, HLA-E, HLA-F, HSPA8, PSME2, TAP1, TAP2* | 11 | 6.10E-05 | Antigen processing and presentation  (hsa04612) | | 2.48E-07 | 11 | *CALR, HLA-A, TAP2, PSME2, HLA-F, HLA-E, HLA-G, B2M, HLA-C, TAP1, HLA-B* | |
| *CCND3, EIF2AK2, HIST1H2BK, HIST1H2BL, HIST1H2BM, HLA-A, HLA-B, HLA-C, HLA-E, HLA-F, IRF9, LYN, NFKBIA, PMAIP1, PXN, SP100* | 16 | 5.27E-04 | Viral carcinogenesis  (hsa05203) | | 6.00E-05 | 13 | *NFKBIA, HIST1H2BM, SP100, LYN, HIST1H2BK, HLA-A, EIF2AK2, IRF9, HLA-F, HLA-E, HLA-G, HLA-C, HLA-B* | |
| *CLDN4, DDX58, EIF2AK1, EIF2AK2, EIF3E, IFIT1, IRF9, NFKBIA, OAS2, OAS3, STAT1, STAT2* | 12 | 1.55E-03 | Hepatitis C  (hsa05160) | | 3.53E-05 | 11 | *EIF3E, NFKBIA, TICAM1, EIF2AK2, OAS3, OAS1, IRF9, STAT1, DDX58, OAS2, IFIT1* | |
| *CCL5, GBP4, IRF9, MFN2, MYD88, NAMPT, NFKBIA, OAS2, OAS3, STAT1, STAT2* | 11 | 0.023708 | NOD-like receptor signaling pathway  (hsa04621) | | 1.26E-05 | 13 | *PANX1, NFKBIA, MYD88, TICAM1, CXCL1, MFN2, BIRC3, OAS3, CCL5, OAS1, IRF9, STAT1, OAS2* | |
| *HLA-A, HLA-B, HLA-C, HLA-DOB, HLA-E, HLA-F* | 6 | 5.68E-03 | Allograft rejection  (hsa05330) | | 2.03E-05 | 7 | *FAS, HLA-A, HLA-F, HLA-E, HLA-G, HLA-C, HLA-B* | |
| *HLA-A, HLA-B, HLA-C, HLA-DOB, HLA-E, HLA-F* | 6 | 7.29E-03 | Graft-versus-host disease  (hsa05332) | | 3.15E-05 | 7 | *FAS, HLA-A, HLA-F, HLA-E, HLA-G, HLA-C, HLA-B* | |
| *HLA-A, HLA-B, HLA-C, HLA-DOB, HLA-E, HLA-F* | 6 | 8.17E-03 | Type I diabetes mellitus  (hsa04940) | | 3.68E-05 | 7 | *FAS, HLA-A, HLA-F, HLA-E, HLA-G, HLA-C, HLA-B* | |
| *CD55, HLA-A, HLA-B, HLA-C, HLA-DOB, HLA-E, HLA-F* | 7 | 7.92E-03 | Viral myocarditis  (hsa05416) | | 2.18E-04 | 7 | *HLA-A, CD55, HLA-F, HLA-E, HLA-G, HLA-C, HLA-B* | |
| *CAPN2, EIF2AK2, GLUD1, H2AFJ, H2AFY, HIST2H2AA4, IRF9, PARP4, STAT1, STAT2, TNFRSF10B, TNFSF10* | 12 | 7.29E-03 | Necroptosis  (hsa04217) | | 8.75E-04 | 10 | *CAPN2, TICAM1, GLUD1, FAS, TNFRSF10B, TNFSF10, EIF2AK2, BIRC3, IRF9, STAT1* | |
| *HLA-A, HLA-B, HLA-C, HLA-DOB, HLA-E, HLA-F* | 6 | 0.021063 | Autoimmune thyroid disease  (hsa05320) | | 1.26E-04 | 7 | *FAS, HLA-A, HLA-F, HLA-E, HLA-G, HLA-C, HLA-B* | |
| *CCND3, EIF2AK2, HES4, HLA-A, HLA-B, HLA-C, HLA-E, HLA-F, IRF9, ISG15, ITGA3, ITGB7, LAMA3, MX1, OASL, PXN, STAT1, STAT2* | 18 | 1.17E-02 | Human papillomavirus infection  (hsa05165) | | 1.81E-04 | 16 | *TICAM1, LAMA3, FAS, HES4, HLA-A, EIF2AK2, IRF9, HLA-F, HLA-E, STAT1, HLA-G, ISG15, OASL, HLA-C, HLA-B, MX1* | |
| *HGS, HLA-A, HLA-B, HLA-C, HLA-DOB, HLA-E, HLA-F, TAP1, TAP2, TUBB3* | 10 | 0.035803 | Phagosome  (hsa04145) | | 5.61E-04 | 10 | *CALR, HLA-A, CD14, TAP2, HLA-F, HLA-E, HLA-G, HLA-C, TAP1, HLA-B* | |
| *B2M, BST2, HLA-A, HLA-B, HLA-C, HLA-E, HLA-F, MYD88, NFKBIA, PXN, TAP1, TAP2* | 12 | 0.044886 | Human immunodeficiency virus 1 infection  (hsa05170) | | 1.44E-07 | 17 | *APOBEC3B, NFKBIA, MYD88, CALR, PAK6, FAS, HLA-A, TAP2, APOBEC3C, BST2, HLA-F, HLA-E, HLA-G, B2M, HLA-C, TAP1, HLA-B* | |
| *EIF2AK2, HLA-A, HLA-B, HLA-C, HLA-E, HLA-F, IRF9, LYN, NFKBIA, STAT1, STAT2* | 11 | 0.045559 | Kaposi sarcoma-associated herpesvirus infection  (hsa05167) | | 6.83E-06 | 14 | *NFKBIA, TICAM1, CXCL1, FAS, LYN, HLA-A, EIF2AK2, IRF9, HLA-F, HLA-E, STAT1, HLA-G, HLA-C, HLA-B* | |

Supplementary table 6. Full list of all KEGG Pathways enriched in differently expressed genes in irradiated (5x2 Gy) HT29 PARP13 KO versus non-irradiated HT29 PARP13 KO cells under 2D cell culture conditions.

| **Category** | **Number of genes** | **FDR** | **Genes** |
| --- | --- | --- | --- |
| Viral myocarditis  (hsa05416) | 11 | 0.036865 | *CAV1, HLA-A, ACTB, CXADR, ITGB2, SGCB, HLA-G, HLA-DMB, EIF4G2, HLA-DQB1, RAC2* |
| Cell adhesion molecules (CAMs)  (hsa04514) | 18 | 0.036865 | *HLA-A, NCAM2, CLDN9, ITGAM, ITGB2, CLDN14, CLDN11, MAG, CLDN24, ICOSLG, HLA-G, CLDN6, CD274, CD99, HLA-DMB, SDC4, HLA-DQB1, OCLN* |
| Leukocyte transendothelial migration (hsa04670) | 15 | 0.038392 | *MAPK11, ACTB, CXCL12, CLDN9, ITGAM, ITGB2, CLDN14, CLDN11, CLDN24, PXN, VAV2, CLDN6, CD99, RAC2, OCLN* |

Additional table 7. Full list of all KEGG Pathways enriched in differently expressed genes in irradiated (5x2 Gy) HT29 PARP13 KO versus non-irradiated HT29 PARP13 KO cells under lr-ECM 3D cell culture conditions.

| **Category** | **Number of genes** | **FDR** | **Genes** |
| --- | --- | --- | --- |
| NF-kappa B signaling pathway  (hsa04064) | 16 | 7.15E-05 | *IL1B, LCK, PTGS2, PLCG2, PLAU, ICAM1, CXCL12, RELB, NFKBIA, LAT, IL1R1, BIRC3, LTB, CXCL2, CCL19, ATM* |
| Human T-cell leukemia virus 1 infection  (hsa05166) | 24 | 0.002287 | *JAK1, HLA-A, WNT11, LCK, ICAM1, RELB, NFKBIA, EGR2, MAPK8, ELK1, TLN1, IL1R1, HLA-G, MYB, PRKACB, NRP1, SLC2A1, MAP2K4, CDC23, HLA-DMB, CSF2, IL2RG, ATM, MYC* |
| TNF signaling pathway  (hsa04668) | 14 | 0.003954 | *IL1B, PTGS2, ICAM1, EDN1, MAP2K6, NFKBIA, CX3CL1, CXCL1, MAPK8, CCL20, BIRC3, MAP2K4, CSF2, CXCL2* |
| Th17 cell differentiation  (hsa04659) | 13 | 0.007832 | *JAK1, IL1B, LCK, IL27RA, NFKBIA, RXRA, MAPK8, LAT, IL1R1, IL23A, HLA-DMB, IL2RG, IFNGR1* |
| Transcriptional misregulation in cancer  (hsa05202) | 18 | 0.007832 | *DOT1L, PLAU, PLAT, ITGAM, ID2, RXRA, PML, MAX, CEBPA, BIRC3, SUPT3H, PAX7, CSF2, DDIT3, RUNX2, ATM, BCL11B, MYC* |
| Leishmaniasis  (hsa05140) | 10 | 0.015814 | *JAK1, IL1B, PTGS2, CYBB, ITGAM, NFKBIA, ELK1, C3, HLA-DMB, IFNGR1* |
| Kaposi sarcoma-associated herpesvirus infection  (hsa05167) | 7 | 0.017105 | *JAK1, HLA-A, PTGS2, PLCG2, ICAM1, MAP2K6, NFKBIA, CXCL1, MAPK8, HLA-G, C3, MAP2K4, CSF2, MICB, CXCL2, MYC, IFNGR1* |
| Legionellosis  (hsa05134) | 8 | 0.029076 | *IL1B, HBS1L, ITGAM, NFKBIA, CXCL1, C3, HSPA6, CXCL2* |

Additional table 8**.** Distribution of cases with low or high change of PARPs expression in tumor tissue after CRT according to demographic and clinicopathological characteristics of the rectal cancer patients.

|  | **Characteristic** | **Change of *PARP9* expression** | |  | **Change of *PARP12* expression** | |  | **Change of *PARP13* expression** | |  | **Change of *PARP14* expression** | |  |
| --- | --- | --- | --- | --- | --- | --- | --- | --- | --- | --- | --- | --- | --- |
|  |  | Low  n (%) | High  n (%) | **p** | Low  n (%) | High  n (%) | **p** | Low  n (%) | High  n (%) | **p** | Low  n (%) | High  n (%) | **p** |
|  | **Age (in years)**  ≤68  >68 | 17 (51.5)  16 (48.5) | 15 (44.1)  19 (55.9) | 0.544 | 15 (48.4)  16 (51.6) | 17 (47.2)  19 (52.8) | >0.999 | 18 (54.5)  15 (45.5) | 14 (41.2)  20 (58.8) | 0.332 | 20 (58.8)  14 (41.2) | 12 (36.4)  21 (63.6) | 0.088 |
|  | **Gender**  Male  Female | 22 (66.7)  11 (33.3) | 20 (58.8)  14 (41.2) | 0.051 | 16 (51.6)  15 (48.4) | 20 (55.6)  16 (44.4) | 0.809 | 22 (66.7)  11 (33.3) | 14 (41.2)  20 (58.8) | 0.051 | 22 (64.7)  12 (35.3) | 14 (42.4)  19 (57.6) | 0.088 |
| **Before treatment** | **Pathological Stage**  I/II  III/IV | 5 (15.2)  28 (84.8) | 2 (5.9)  32 (94.1) | 0.259 | 3 (9.7)  28 (90.3) | 4 (11.1)  32 (88.9) | >0.999 | 3 (9.1)  30 (90.9) | 4 (11.8)  30 (88.2) | >0.999 | 4 (11.8)  30 (88.2) | 3 (9.1)  30 (90.9) | >0.999 |
|  | **Primary tumor status**  T2/3  T4 | 28 (84.8)  5 (15.2) | 28 (82.4)  6 (17.6) | >0.999 | 25 (80.6)  6 (19.4) | 31 (86.1)  5 (13.9) | 0.742 | 27 (81.8)  6 (18.2) | 29 (85.3)  5 (14.7) | 0.752 | 29 (85.3)  5 (14.7) | 27 (81.8)  6 (18.2) | 0.752 |
|  | **Lymph node status**  N0  N1-2 | 1 (3.0)  32 (97.0) | 3 (8.8)  31 (91.2) | 0.614 | 0 (0)  31 (100) | 4 (11.1)  32 (88.9) | 0.118 | 1 (3.0)  32 (97.0) | 3 (8.8)  31 (91.2) | 0.614 | 2 (5.9)  32 (94.1) | 2 (6.1)  31 (93.9) | >0.999 |
| **After treatment** | **Primary tumor status**  T0/1/2  T3/4 | 13 (39.4)  20 (60.6) | 14 (41.2)  20 (58.8) | >0.999 | 14 (45.2)  17 (54.8) | 13 (36.1)  23 (63.9) | 0.467 | 11 (33.3)  22 (66.7) | 16 (47.1)  18 (52.9) | 0.322 | 14 (41.2)  20 (58.8) | 13 (39.4)  20 (60.6) | >0.999 |
|  | **Lymph node status**  N0  N1-2 | 22 (66.7)  11 (33.3) | 21 (61.8)  13 (38.2) | 0.800 | 24 (77.4)  7 (22.6) | 19 (52.8)  17 (47.2) | **0.044** | 21 (63.6)  12 (36.4) | 22 (64.7)  12 (35.3) | >0.999 | 22 (64.7)  12 (35.3) | 21 (63.6)  12 (36.4) | >0.999 |
|  | **Time after CRT to surgery**  8 weeks  12 weeks | 18 (54.5)  15 (45.5) | 12 (35.3)  22 (64.7) | 0.144 | 13 (41.9)  18 (58.1) | 17 (47.2)  19 (52.8) | 0.806 | 19 (57.6)  14 (42.4) | 11 (32.4)  23 (67.6) | 0.051 | 19 (44.8)  15 (55.2) | 11 (33.3)  22 (66.7) | 0.087 |
|  | **Dworak tumor regression grading**  1  2/3/4 | 9 (27.3)  24 (72.7) | 8 (23.5)  26 (76.5) | 0.784 | 11 (35.5)  20 (64.5) | 6 (16.7)  30 (83.3) | 0.096 | 9 (27.3)  24 (72.7) | 8 (23.5)  26 (76.5) | 0.784 | 10 (29.4)  24 (70.6) | 7 (21.2)  26 (78.8) | 0.576 |
|  | **Response status**  Progression  Stable disease | 9 (27.3)  24 (72.7) | 8 (23.5)  26 (76.5) | 0.784 | 9 (29.0)  22 (71.0) | 8 (22.2)  28 (77.8) | 0.581 | 8 (24.2)  25 (75.8) | 9 (73.5)  25 (26.5) | >0.999 | 9 (26.5)  25 (73.5) | 8 (24.2)  25 (75.8) | >0.999 |

Additional table 9. Distribution of cases with low or high change of PARPs expression in normal tissue after CRT according to demographic and clinicopathological characteristics of the rectal cancer patients.

|  | **Characteristic** | **Change of *PARP9* expression** | |  | **Change of *PARP12* expression** | |  | **Change of *PARP13* expression** | |  | **Change of *PARP14* expression** | |  |
| --- | --- | --- | --- | --- | --- | --- | --- | --- | --- | --- | --- | --- | --- |
|  |  | Low  n (%) | High  n (%) | **p** | Low  n (%) | High  n (%) | **p** | Low  n (%) | High  n (%) | **p** | Low  n (%) | High  n (%) | **p** |
|  | **Age (in years)**  ≤68  >68 | 13 (65.0)  7 (35.0) | 5 (45.5)  6 (54.5) | 0.449 | 10 (62.5)  6 (37.5) | 8 (53.3)  7 (46.7) | 0.722 | 11 (68.8)  5 (31.2) | 7 (46.7)  8 (53.3) | 0.285 | 11 (68.7)  5 (31.3) | 7 (46.7)  8 (53.3) | 0.285 |
|  | **Gender**  Male  Female | 11 (55.0)  9 (45.0) | 6 (54.5)  5 (45.5) | >0.999 | 10 (62.5)  6 (37.5) | 7 (46.7)  8 (53.3) | 0.479 | 10 (62.5)  6 (37.5) | 7 (46.7)  8 (53.3) | 0.479 | 11 (68.7)  5 (31.3) | 6 (40.0)  9 (60.0) | 0.156 |
| **Before treatment** | **Pathological Stage**  I/II  III/IV | 3 (15.0)  17 (85.0) | 2 (18.2)  9 (81.8) | >0.999 | 1 (6.3)  15 (93.8) | 4 (26.7)  11 (73.3) | 0.172 | 3 (18.8)  13 (81.3) | 2 (13.3)  13 (86.7) | >0.999 | 1 (6.3)  15 (93.7) | 4 (26.7)  11 (73.3) | 0.172 |
|  | **Primary tumor status**  T2/3  T4 | 19 (95.0)  1 (5.0) | 9 (81.8)  2 (18.2) | 0.281 | 15 (93.8)  1 (6.3) | 13 (86.7)  2 (13.3) | 0.600 | 14 (87.5)  2 (12.5) | 14 (93.3)  1 (6.7) | >0.999 | 15 (93.7)  1 (6.3) | 13 (86.7)  2 (13.3) | 0.600 |
|  | **Lymph node status**  N0  N1-2 | 4 (20.0)  16 (80.0) | 0 (0.0)  11 (100) | 0.269 | 2 (12.5)  14 (87.5) | 2 (13.3)  13 (86.7) | >0.999 | 4 (25.0)  12 (75.0) | 0 (0.0)  15 (100) | 0.101 | 3 (18.8)  13 (81.2) | 1 (6.7)  14 (93.3) | 0.600 |
| **After treatment** | **Primary tumor status**  T0/1/2  T3/4 | 8 (40.0)  12 (60.0) | 2 (18.2)  9 (81.8) | 0.262 | 8 (50.0)  8 (50.0) | 2 (13.3)  13 (86.7) | 0.054 | 4 (25.0)  12 (75.0) | 6 (40.0)  9 (60.0) | 0.458 | 5 (31.3)  11 (68.7) | 5 (33.3)  10 (66.7) | >0.999 |
|  | **Lymph node status**  N0  N1/2 | 13 (65.0)  7 (35.0) | 7 (63.6)  4 (36.4) | >0.999 | 10 (62.5)  6 (37.5) | 10 (66.7)  5 (33.3) | >0.999 | 10 (62.5)  6 (37.5) | 10 (66.7)  5 (33.3) | >0.999 | 8 (50.0)  8 (50.0) | 12 (80.0)  3 (20.0) | 0.135 |
|  | **Time after CRT to surgery**  8 weeks  12 weeks | 11 (55.0)  9 (45.0) | 6 (54.5)  5 (45.5) | >0.999 | 8 (50.0)  8 (50.0) | 9 (60.0)  6 (40.0) | 0.722 | 8 (50.0)  8 (50.0) | 9 (60.0)  6 (40.0) | 0.722 | 11 (68.7)  5 (31.3) | 6 (40.0)  9 (60.0) | 0.156 |
|  | **Dworak tumor regression grade**  1  2/3/4 | 4 (20.0)  16 (80.0) | 6 (54.5)  5 (45.5) | 0.106 | 6 (37.5)  10 (62.5) | 4 (26.7)  11 (73.3) | 0.704 | 5 (31.3)  11 (68.7) | 5 (33.3)  10 (66.7) | >0.999 | 4 (25.0)  12 (75.0) | 6 (40.0)  9 (60.0) | 0.458 |
|  | **Response status**  Progression  Stable disease | 6 (30.0)  14 (70.0) | 3 (27.3)  8 (72.7) | >0.999 | 4 (25.0)  12 (75.0) | 5 (33.3)  10 (66.7) | 0.704 | 5 (31.3)  11 (68.7) | 4 (26.7)  11 (73.3) | 0.546 | 4 (25.0)  12 (75.0) | 5 (33.3)  10 (66.7) | 0.704 |

Additional table 10. Univariate and Multivariate Cox regression of prognostic factors for rectal cancer patients’ OS in tumor tissue.

|  | **Variables** | **Univariate** | | **Multivariate for *PARP9* expression** | | **Multivariate for *PARP12* expression** | | **Multivariate for *PARP13* expression** | | **Multivariate for *PARP14* expression** | |
| --- | --- | --- | --- | --- | --- | --- | --- | --- | --- | --- | --- |
|  |  | HR (95% CI) | p-value | HR (95% CI) | p-value | HR (95% CI) | p-value | HR (95% CI) | p-value | HR (95% CI) | p-value |
|  | **Age (in years)**  ≤68  >68 | Ref.  0.400 (0.126-1.272) | 0.121 | Ref.  1.171 (0.226-6.074) | 0.851 | Ref.  0.519 (0.132-2.036) | 0.347 | Ref.  0.532 (0.138-2.051) | 0.359 | Ref.  1.054 (0.256-4.338) | 0.942 |
|  | **Gender**  Male  Female | Ref.  0.497 (0.152-1.621) | 0.246 | Ref.  0.665 (0.174-2.543) | 0.551 | Ref.  0.374 (0.096-1.461) | 0.157 | Ref.  0.540 (0.139-2.089) | 0.372 | Ref.  0.276 (0.067-1.139) | 0.075 |
| **Before treatment** | **Primary tumor status**  T2/3  T4 | Ref.  0.867 (0.194-3.978) | 0.867 | Ref.  0.905 (0.163-5.024) | 0.909 | Ref.  1.020 (0.199-5.236) | 0.982 | Ref.  0.820 (0.159-4.225) | 0.812 | Ref.  0.522 (0.094-2.887) | 0.456 |
| **After**  **treatment** | **Primary tumor status**  T0/1/2  T3/4 | Ref.  10.629 (1.373-82.265) | **0.024** | Ref.  7.728 (0.767-77.825) | 0.083 | Ref.  7.115 (0.703-71.991) | 0.097 | Ref.  6.058 (0.640-57.388) | 0.116 | Ref.  20.592 (1.697-249.819) | **0.018** |
|  | **Lymph node status**  N0  N1-2 | Ref.  10.844 (2.402-48.963) | **0.002** | Ref.  10.292 (1.684-62.915) | **0.012** | Ref.  8.095 (1.521-43.084) | **0.014** | Ref.  7.120 (1.502-33.755) | **0.013** | Ref.  11.588 (1.879-71.479) | **0.008** |
|  | **Time after CRT to surgery**  12 weeks  8 weeks | Ref.  1.347 (0.448-4.046) | 0.596 | Ref.  0.513 (0.155-1.691) | 0.272 | Ref.  0.380 (0.101-1.425) | 0.151 | Ref.  0.443 (0.141-1.392) | 0.163 | Ref.  0.148 (0.031-0.707) | **0.017** |
|  | **Dworak tumor regression grade**  2/3/4  1 | Ref.  3.310 (1.110-9.868) | **0.032** | Ref.  1.571 (0.415-5.949) | 0.506 | Ref.  1.641 (0.448-6.007) | 0.455 | Ref.  1.888 (0.563-6.334) | 0.303 | Ref.  1.831 (0.462-7.260) | 0.390 |
|  | **Change of *PARP9* expression**  Low  High | Ref.  0.252 (0.068-0.930) | **0.039** | Ref.  0.147 (0.025-0.877) | **0.035** | - | - | - | - | - | - |
|  | **Change of *PARP12* expression**  Low  High | Ref.  1.085 (0.363-3.239) | 0.884 | - | **-** | Ref.  0.542 (0.143-2.061) | 0.369 | - | - | - | - |
|  | **Change of *PARP13* expression**  Low  High | Ref.  0.396 (0.122-1.289) | 0.124 | - | **-** | - | - | Ref.  0.443 (0.141-1.392) | 0.163 | - | - |
|  | **Change of *PARP14* expression**  Low  High | Ref.  0.253 (0.069-0.930) | **0.039** | - | *-* | - | - | - | - | Ref.  **0.074 (0.012-0.457)** | **0.005** |

Additional table S11. Univariate and Multivariate Cox regression of prognostic factors for rectal cancer patients’ OS in tumor tissue.

|  | **Variables** | **Univariate** | | **Multivariate for *OAS2* expression** | | **Multivariate for *IFITM1* expression** | | **Multivariate for *IFIT1*  expression** | |
| --- | --- | --- | --- | --- | --- | --- | --- | --- | --- |
|  |  | HR (95% CI) | p-value | HR (95% CI) | p-value | HR (95% CI) | p-value | HR (95% CI) | p-value |
|  | **Age (in years)**  ≤68  >68 | Ref.  0.400 (0.126-1.272) | 0.121 | Ref.  0.724 (0.170-3.081) | 0.662 | Ref.  0.627 (0.142-2.765) | 0.537 | Ref.  0.639 (0.136-3.006) | 0.571 |
|  | **Gender**  Male  Female | Ref.  0.497 (0.152-1.621) | 0.246 | Ref.  0.424 (0.106-1.701) | 0.226 | Ref.  0.508 (0.102-2.538) | 0.409 | Ref.  0.416 (0.102-1.692) | 0.220 |
| **Before treatment** | **Primary tumor status**  T2/3  T4 | Ref.  0.867 (0.194-3.978) | 0.867 | Ref.  1.039 (0.199-5.432) | 0.964 | Ref.  0.957 (0.181-5.060) | 0.959 | Ref.  0.882 (0.155-5.016) | 0.888 |
| **After**  **treatment** | **Primary tumor status**  T0/1/2  T3/4 | Ref.  10.629 (1.373-82.265) | **0.024** | Ref.  6.562 (0.661-65.192) | 0.108 | Ref.  5.971 (0.611-58.334) | 0.124 | Ref.  6.219 (0.647-59.762) | 0.113 |
|  | **Lymph node status**  N0  N1-2 | Ref.  10.844 (2.402-48.963) | **0.002** | Ref.  8.004 (1.566-40.915) | **0.012** | Ref.  8.374 (1.417-49.499) | **0.019** | Ref.  7.110 (1.425-35.481) | **0.017** |
|  | **Time after CRT to surgery**  12 weeks  8 weeks | Ref.  1.347 (0.448-4.046) | 0.596 | Ref.  0.666 (0.177-2.504) | 0.547 | Ref.  0.512 (0.130-1.871) | 0.311 | Ref.  0.536 (0.150-1.917) | 0.337 |
|  | **Dworak tumor regression grade**  2/3/4  1 | Ref.  3.310 (1.110-9.868) | **0.032** | Ref.  1.142 (0.281-4.650) | 0.853 | Ref.  1.172 (0.261-5.261) | 0.836 | Ref.  1.405 (0.372-5.308) | 0.616 |
|  | **Change of *OAS2* expression**  Low  High | Ref.  0.476 (0.143-1.586) | 0.227 | Ref.  0.475 (0.117-1.924) | 0.297 | - | - | - | - |
|  | **Change of *IFITM1* expression**  Low  High | Ref.  1.268 (0.405-3.968) | 0.683 | - | - | Ref.  0.561 (0.098-3.197) | 0.515 | - | - |
|  | **Change of *IFIT1* expression**  Low  High | Ref.  1.605 (0.507-5.080) | 0.421 | - | - | - | - | Ref.  0.732 (0.167-3.201) | 0.679 |
