# Supplementary material for: PARP9-PARP13-PARP14 axis tunes colorectal cancer response to radiotherapy
Source: J Exp Clin Cancer Res. 2025 Jul 11;44:199. doi: 10.1186/s13046-025-03439-y (PMC12247367; doi:10.1186/s13046-025-03439-y)
Supplement: Supplementary file 2 — Supplementary Material 2 [file 13046_2025_3439_MOESM2_ESM.docx]

**
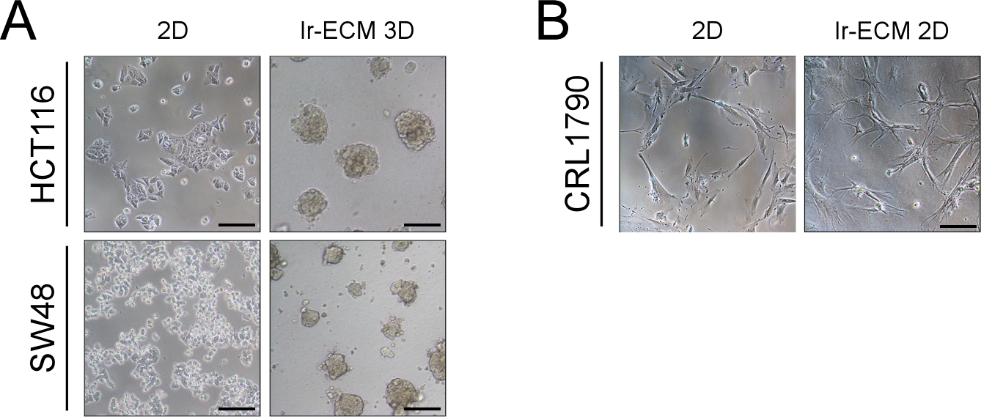
**

**Additional figure 1.** (**A**) Representative phase-contrast images of colorectal cancer cells grown under two different plating conditions: monolayer culture (2D) and three-dimensional laminin rich-extracellular matrix culture (lr-ECM 3D). (**B**) Representative phase-contrast images of normal colon cells grown under two different plating conditions: monolayer culture (2D) and two-dimensional laminin rich-extracellular matrix culture (lr-ECM 2D). Scale bars indicate 200 µm.


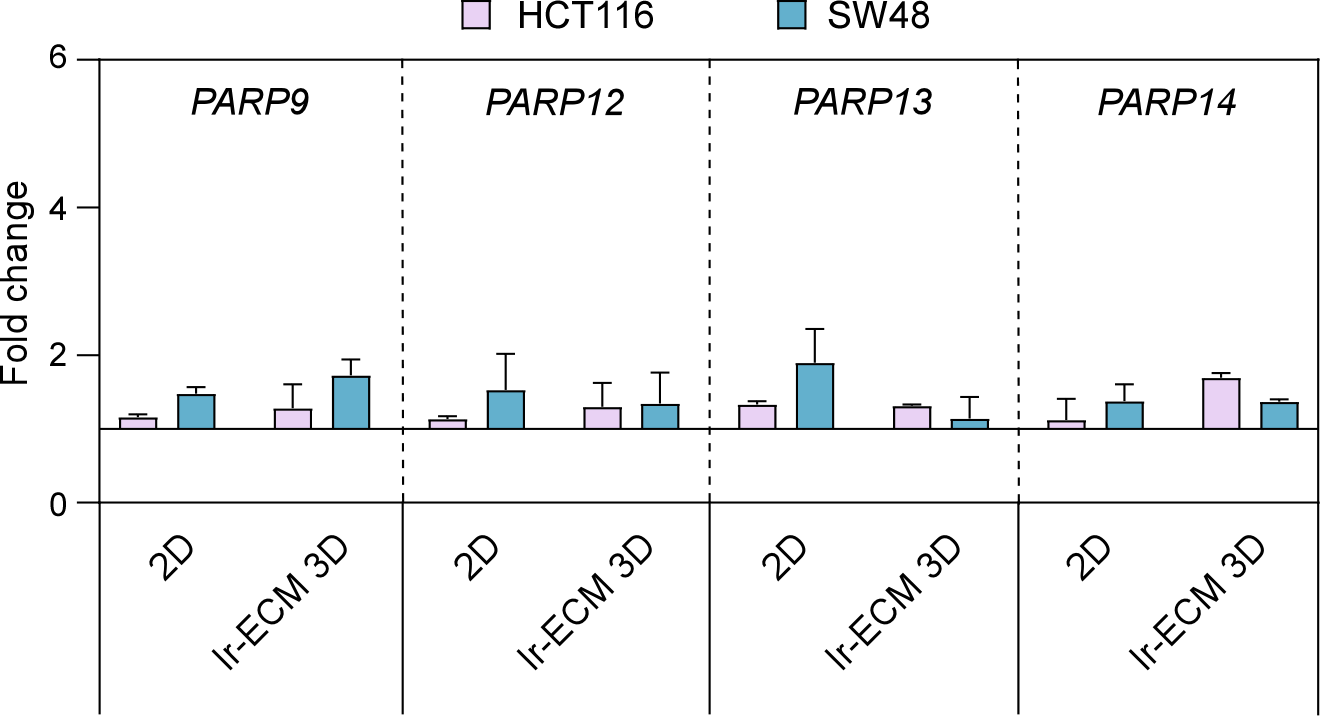


**Additional figure 2.** Expression of PARP9,12,13,14 genes was examined using RT-qPCR in colorectal cancer cells (HCT116, SW48) cultivated under 2D or lr-ECM 3D cell culture conditions after exposure to multifractionated irradiation (5x2 Gy). Results show means with error bars representing standard deviation (n=3, p>0.05, Student’s t-test).

**
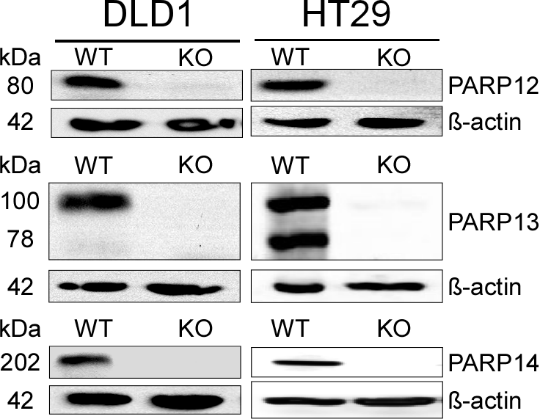
**

**Additional figure 3.** DLD1 and HT29 cell sublines with indicated PARP knockouts were generated using CRISPR/Cas9 genome editing. The knockouts were confirmed through western blot analysis as shown in representative images.

**
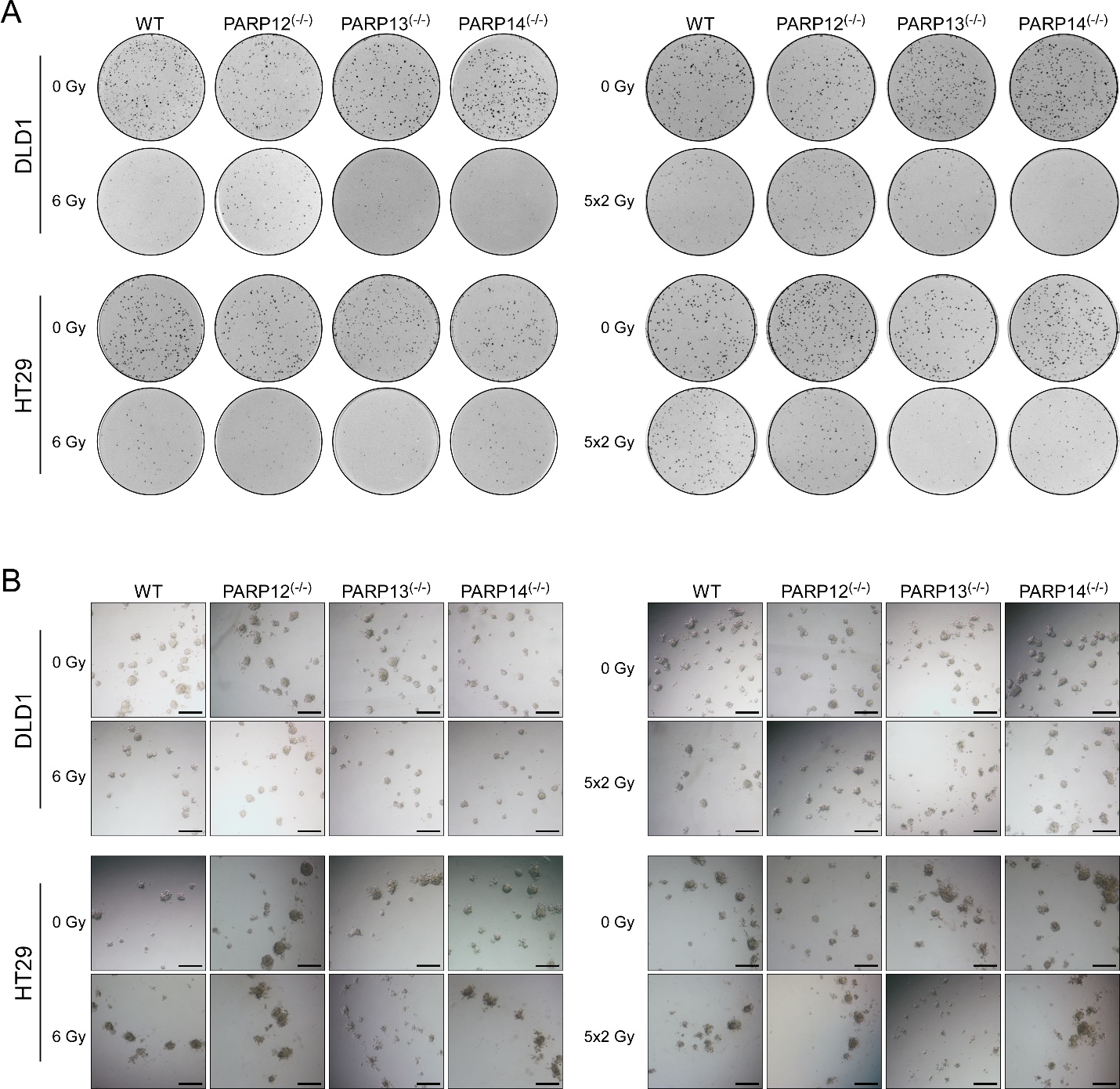
**

**Additional figure 4.** Representative images of colony formation assays of DLD1 and HT29 cells in (A) 2D and (B) lr-3D culture models. Scale bars indicate 200 µm.


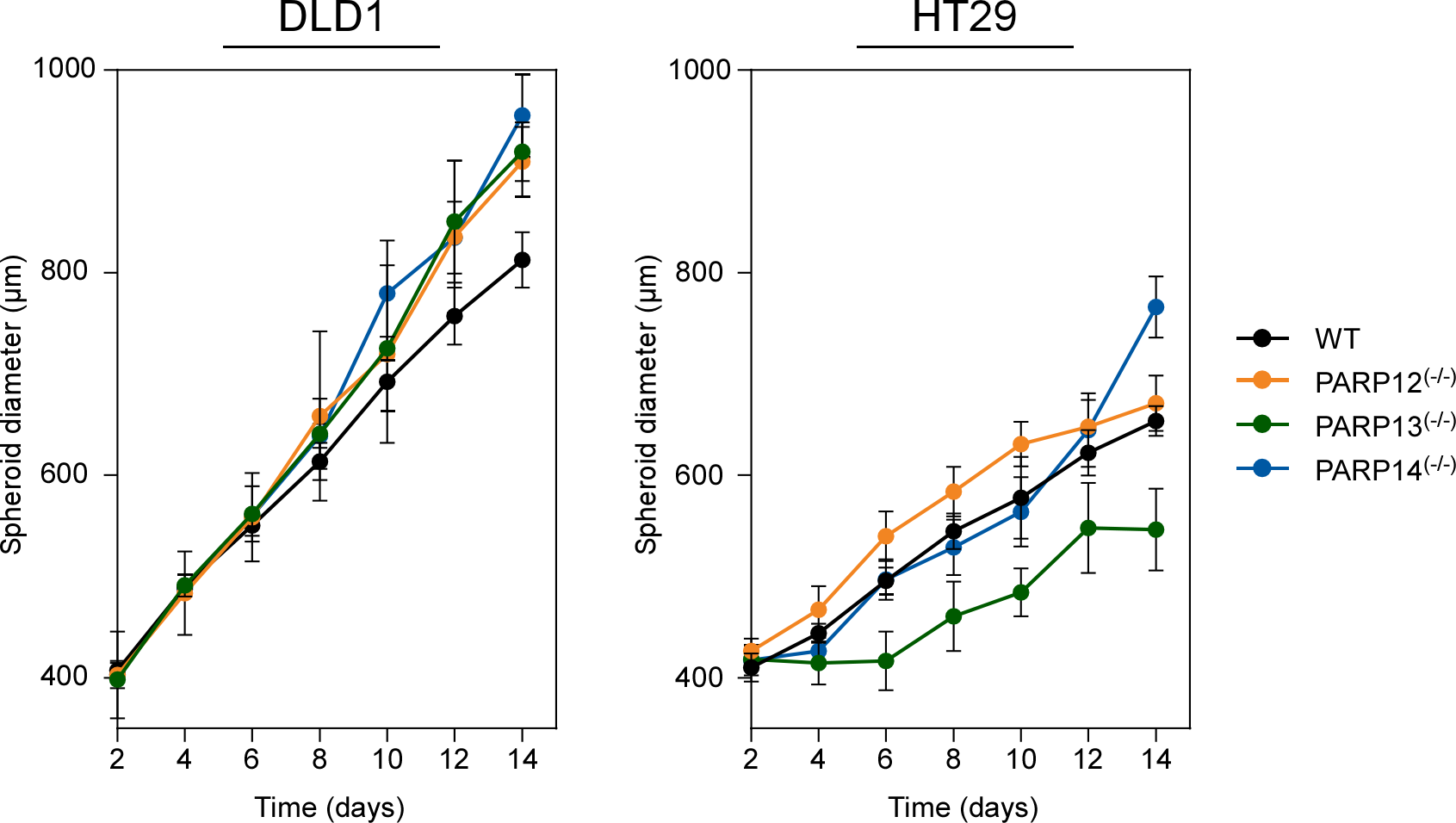


**Additional figure 5.** Control spheroid growth curves. Results show means with error bars representing standard deviation (n=3).


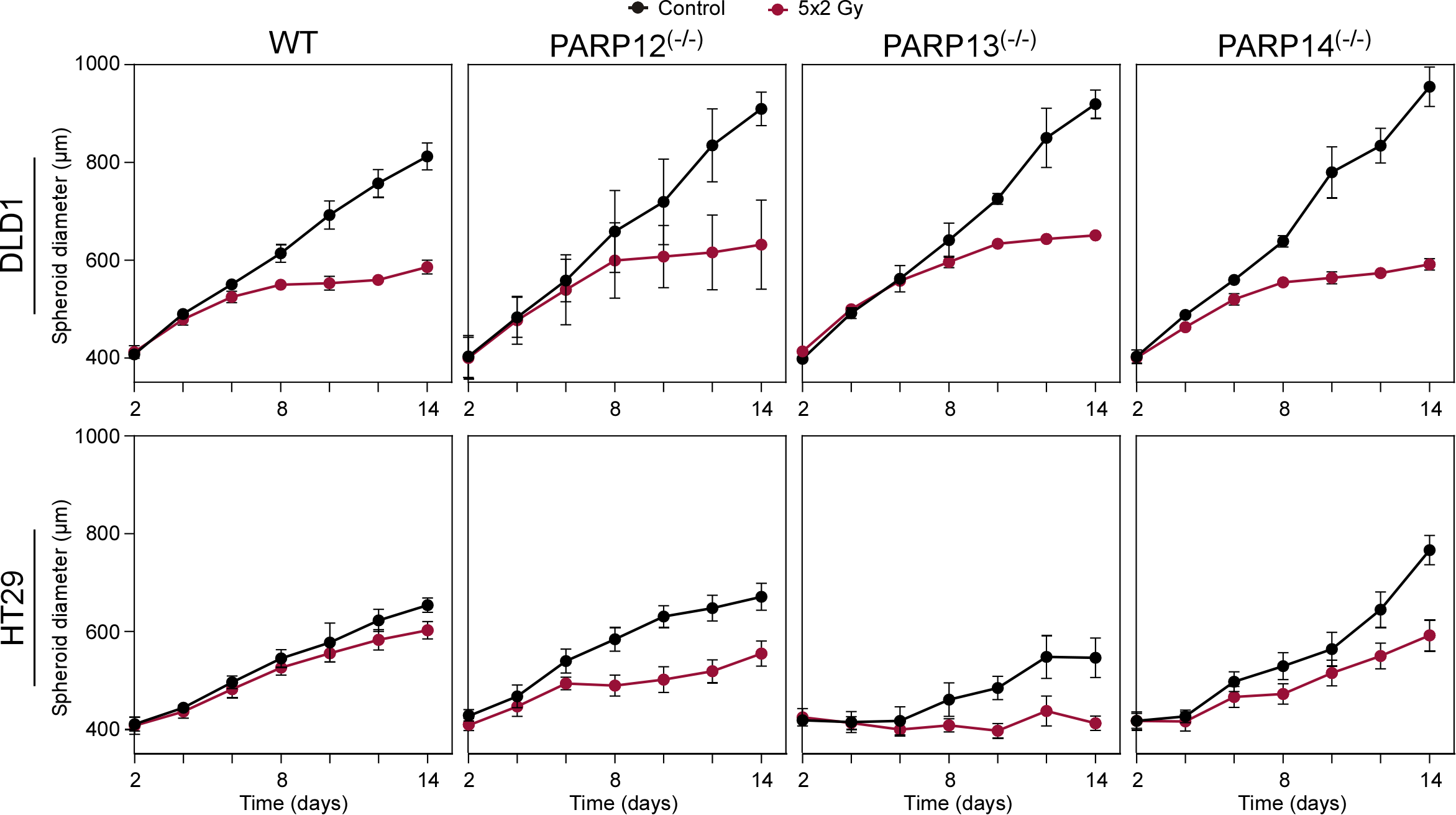


**Additional figure 6.** Spheroid growth curves. MCS were irradiated with a multifractionated dose (5x2 Gy) regimen, and their diameter was measured every two days. Results show means with error bars representing standard deviation (n=3).

**
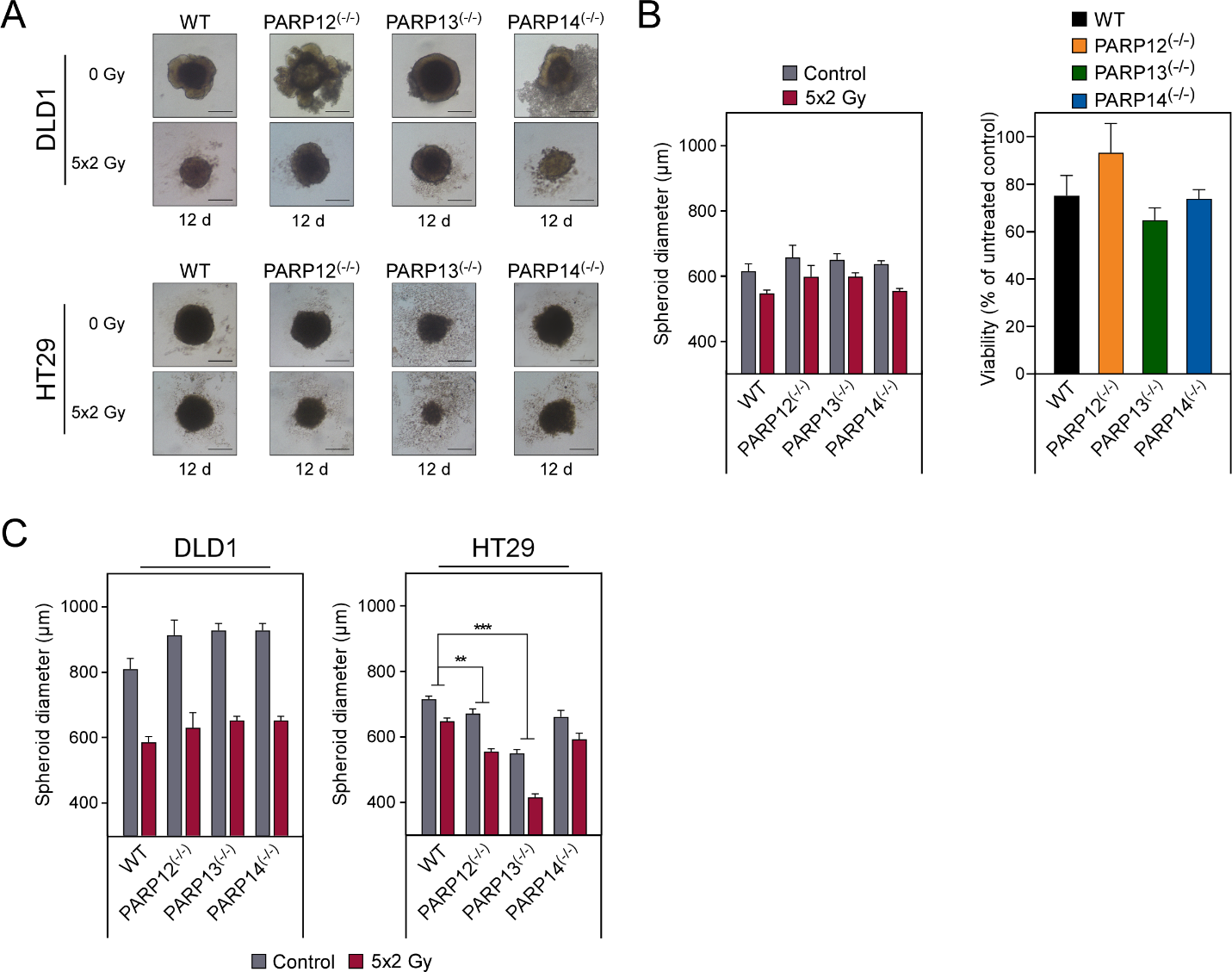
**

**Additional figure 7.**  (**A**) Morphology of CRC PARP knockout cell spheroids and (**C**) their growth kinetics after multifractionated irradiation treatment. Representative images show the spheroids grown at the optimal seeding densities on day 12 after the first irradiation treatment. Scale bars indicate 200 µm. Growth kinetics are represented by spheroid diameter on day 12 after the first irradiation treatment. Non-irradiated spheroids were used as controls. (**B**) Spheroid growth kinetics and viability of DLD1 PARP knockout cells following multifractionated irradiation treatment. Spheroid growth is represented by spheroid diameter on day 6 after the dose of irradiation; same time point for MCS viability. Non-irradiated spheroids were used as the control. Results show means with error bars representing standard deviation (n=3, **p<0.01, ***p<0.001, two-way ANOVA, Student’s t-test).


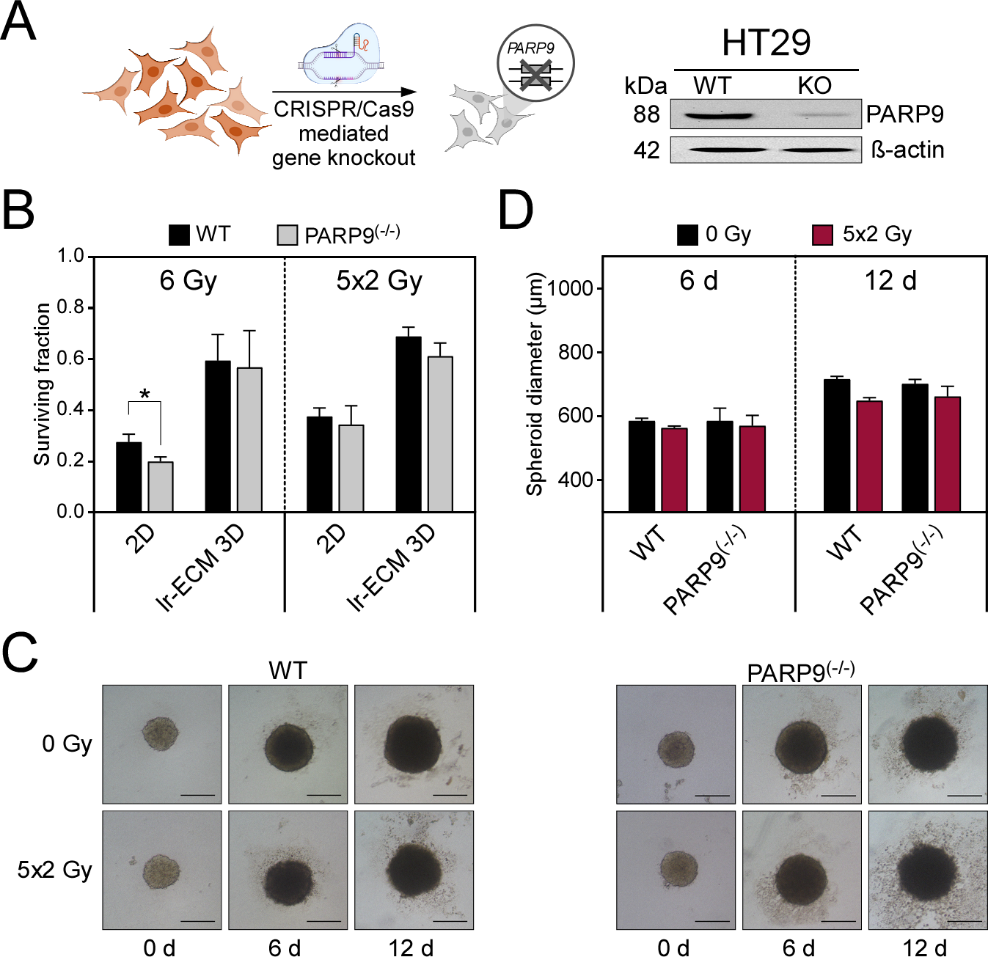


**Additional figure 8.** (**A**) HT29 cell subline with PARP9 partial knockout generated using CRISPR/Cas9 genome editing. The partial knockout was confirmed by western blot analysis as shown in representative image. (**B**) Clonogenic survival of HT29 PARP9 knockout cells after irradiation with a single dose (6 Gy) or multifractionated (5x2 Gy) regimens. Wild type cells were used as the control. Results show means with error bars representing standard deviation (n=3, **p*<0.05, Student’s t-test). (**C**) Morphology of HT29 PARP9 knockout cells spheroids and (**D**) their growth kinetics following multifractionated irradiation treatment. Representative images show the spheroids grown at the optimal seeding densities between day 0 and 12. Scale bars indicate 200 µm. The growth kinetics are represented by spheroid diameter on the 6^th^ and 12^th^ day after the first irradiation. Unirradiated spheroids were used as the control. Results show means with error bars representing standard deviation (n=3, no statistically significant changes, two-way ANOVA).

**
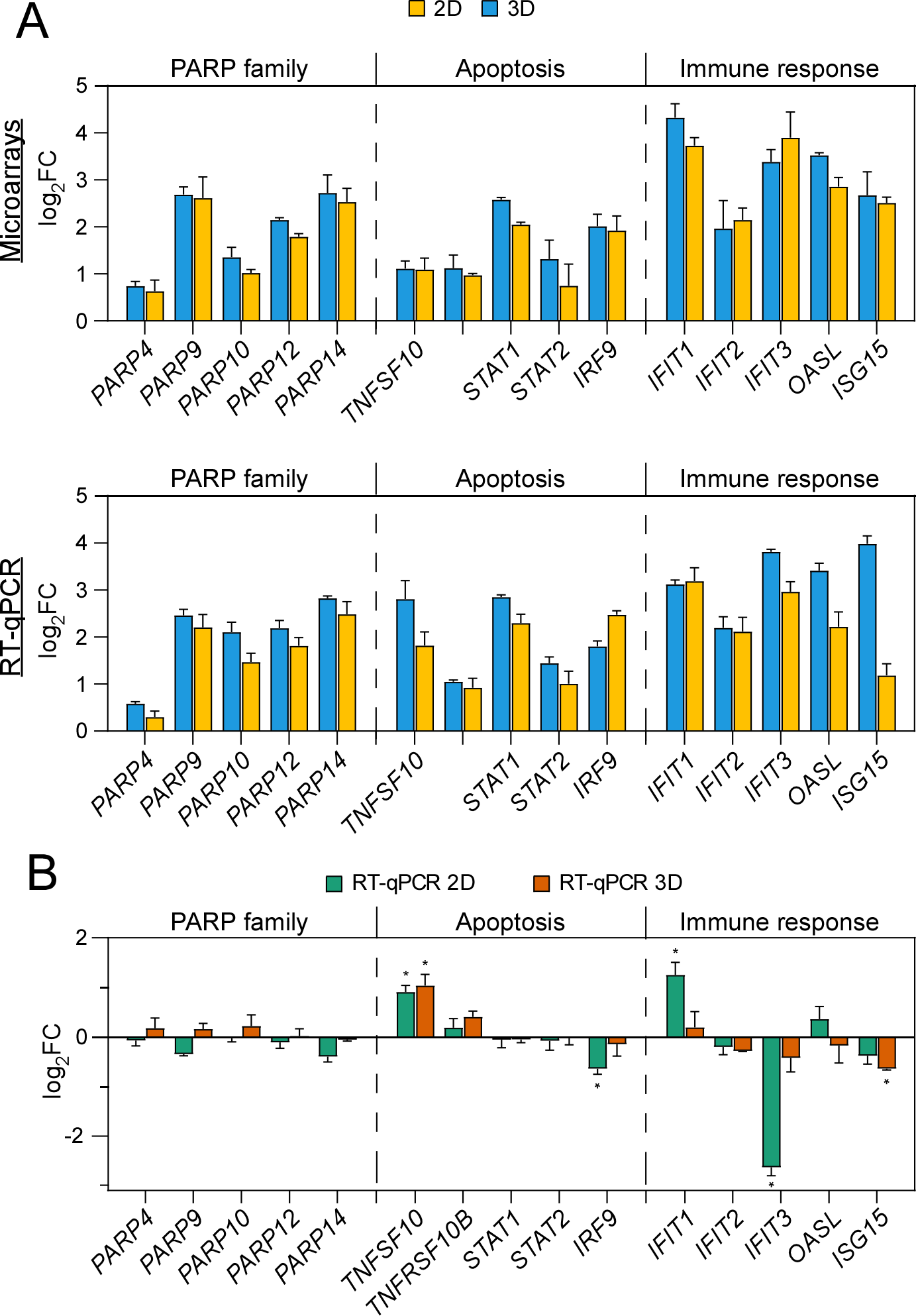
**

**Additional figure 9.** (**A**) Microarray data validation in HT29 PARP13 KO cells by RT-qPCR. Bars indicate fold change of representative deregulated genes belonging to three distinct groups: PARP family, apoptosis, and immune response in DLD1 PARP13 knockout cells compared to control wild type cells. The results display means with error bars representing standard deviation (*n*=3, **p*<0.05, Student’s t-test). (**B**) Microarray data validation using RT-qPCR analysis in DLD1 PARP13 KO cells. Bars indicate fold change of representative deregulated genes belonging to three distinct groups: PARP family, apoptosis, and immune response in DLD1 PARP13 knockout cells compared to control wild type cells. The results display means with error bars representing standard deviation (*n*=3, **p*<0.05, Student’s t-test).


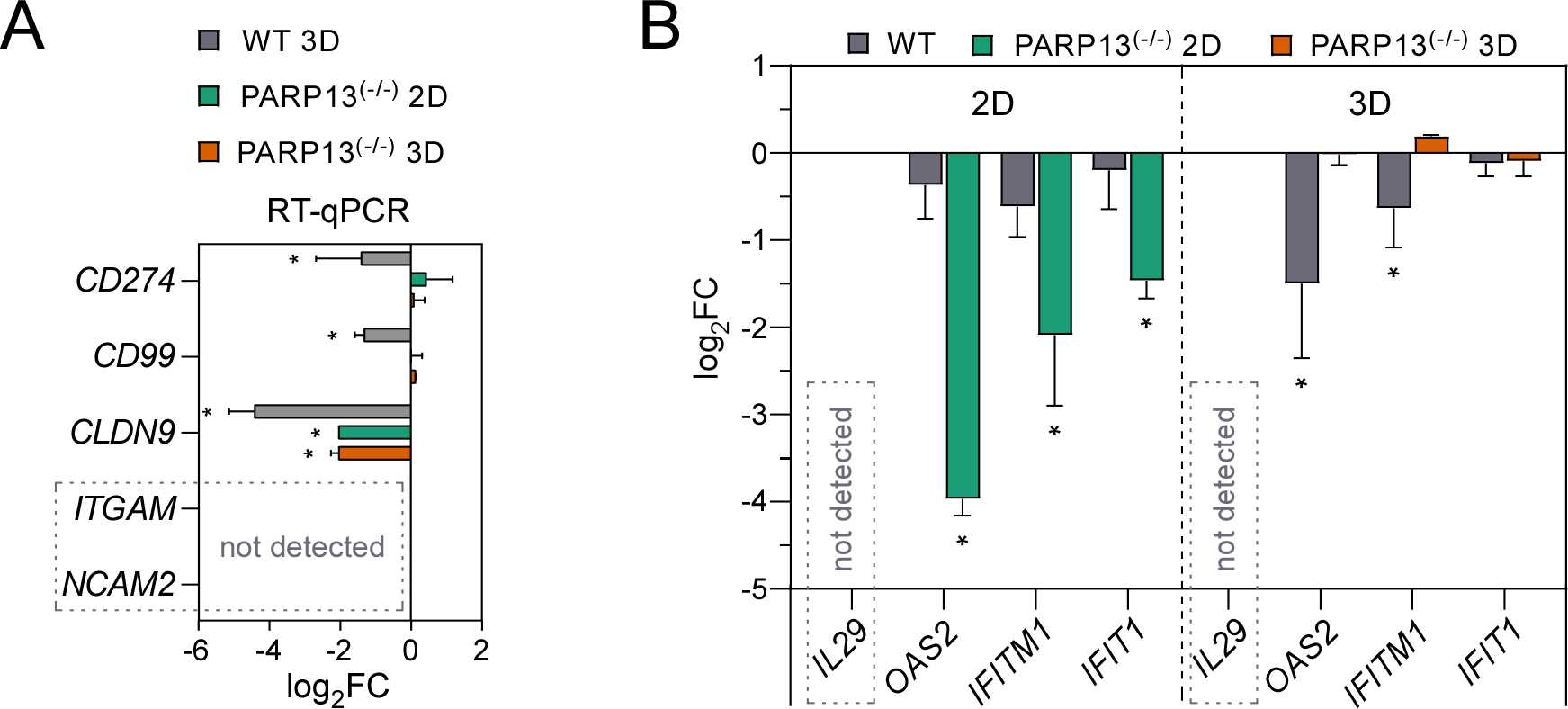


**Additional figure 10.** (**A**) Microarray data validation in DLD1 PARP13 KO cells by RT-qPCR. Results are presented as means with error bars representing standard deviation (*n*=3, **p*<0.05, Student’s t-test). (**B**) Confirmation of differential expression of selected four immune response-related genes in DLD1 wild type and PARP13 knockout irradiated cells using RT-qPCR. Results demonstrate means (*n*=3, **p*<0.05, Student’s t-test).


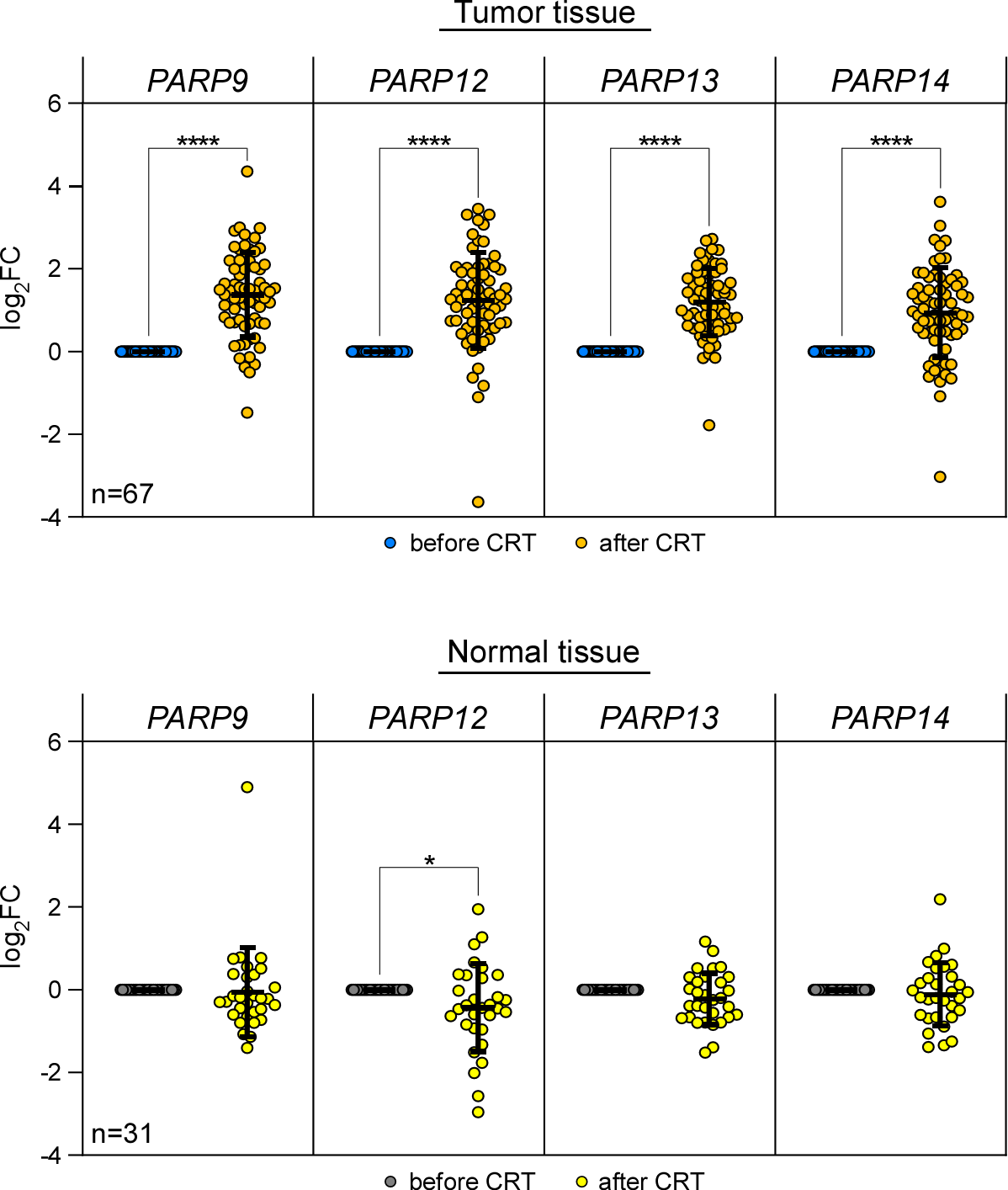


**Additional figure 11.** qRT-PCR analysis of PARP gene expression changes after CRT in tumor and normal tissue sample groups. The cycle threshold (Ct) values of target genes were normalized to GAPDH, ACT and TBP levels, fold change before treatment was set to 1.0. Lines within boxes indicate gene fold change mean values, while whiskers denote standard deviation of the gene fold change values (tumor n=67, normal n=31, Student’s t test, *p < 0.05 and ****p < 0.0001).


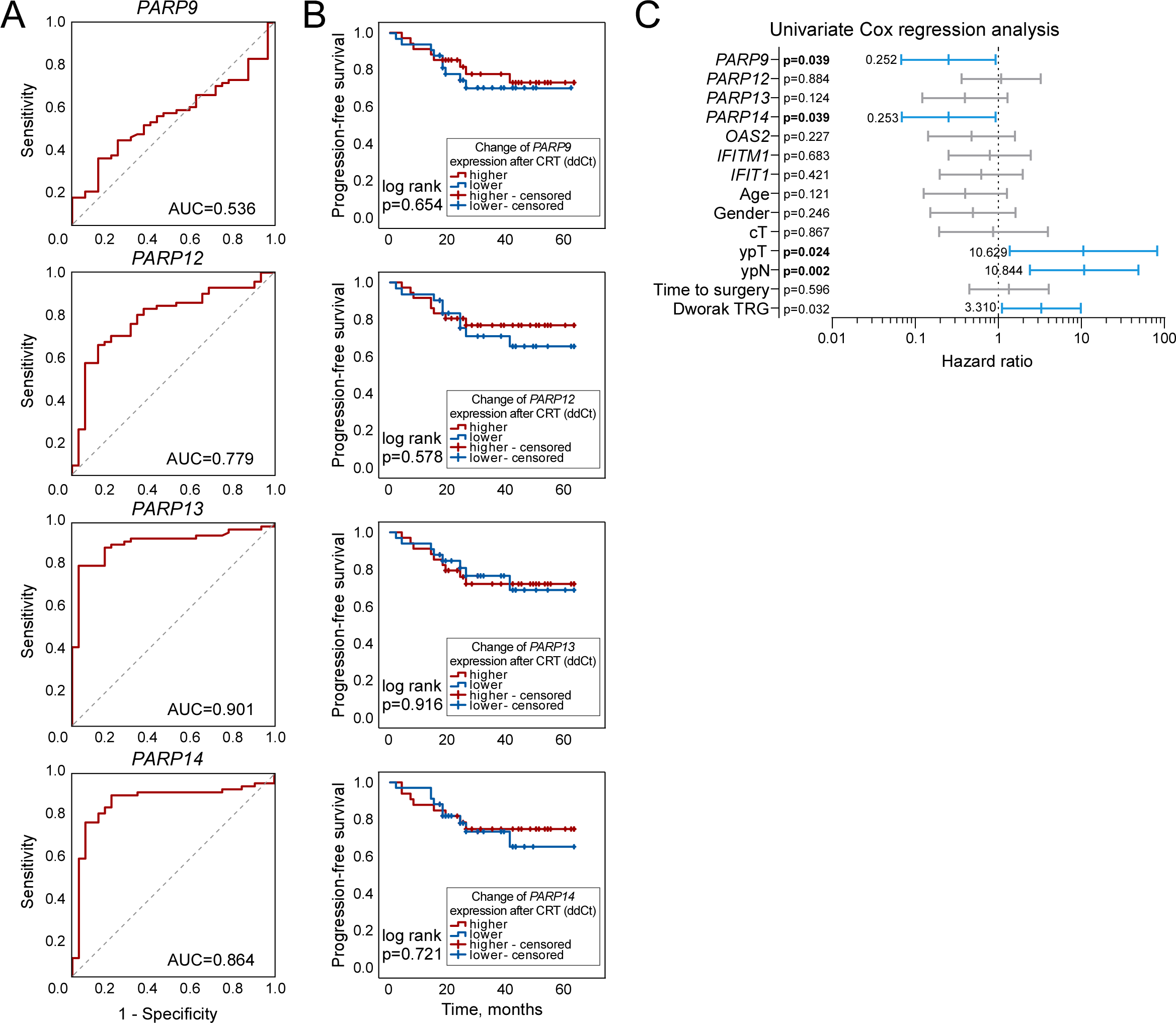


**Additional figure 12**. (**A**) Diagnostic ROC curve analysis showing sensitivity and specificity of *PARP9, PARP12*, *PARP13*, and *PARP14* before CRT. AUC denotes the area under the ROC curve. (**B**) Kaplan-Meier survival curves demonstrating the association between changes in PARP expression after CRT and progression-free (PFS) in rectal cancer samples (n=67). Patients were stratified into high and low change of expression groups according to the mean value. Curves were compared using the log-rank test, *p* values shown. (**C**) Prognostic performance of PARP genes expression changes and clinicopathologic features by univariate Cox regression analysis. Forest plot illustrates the hazard ratio (vertical bar and number above it) and 95% confidence intervals (whiskers) associated with predictors for rectal cancer patients’ OS in tumor samples (n=67). Significant predictors are highlighted in blue, with displayed *p* values.


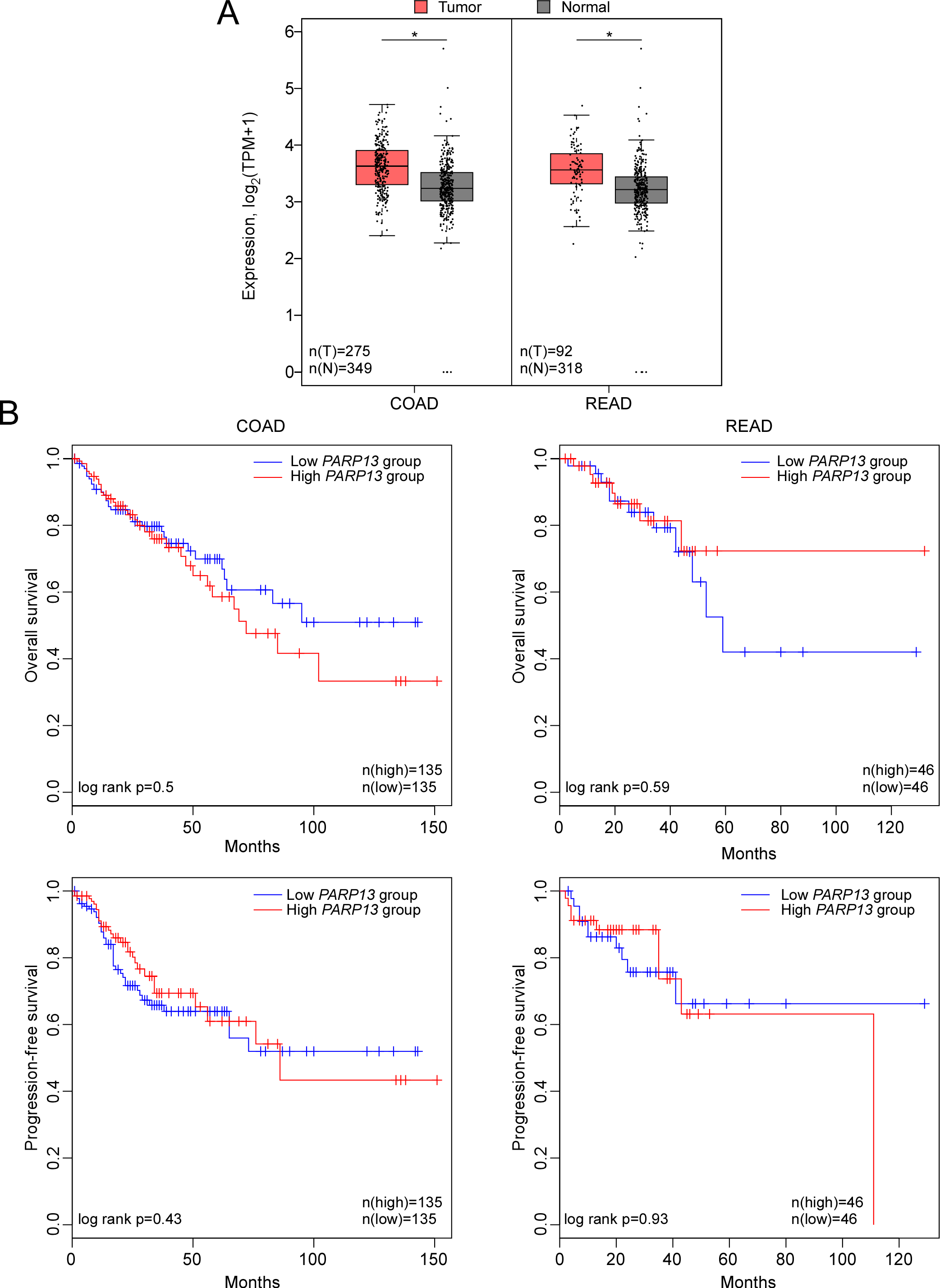


**Additional figure 13.** (**A**) PARP13 expression in colorectal (COAD) and rectal (READ) adenocarcinoma patient tumor and normal tissues. (**B**) Kaplan-Meier survival curves demonstrating the association between low and high PARP13 expression and overall survival or progression-free survival in COAD and READ patient samples. Patients were stratified into high and low expression groups according to the median value. Curves were compared using the log-rank test, *p* values are shown. Data generated from open TCGA and GTEx data using GEPIA2 tool.


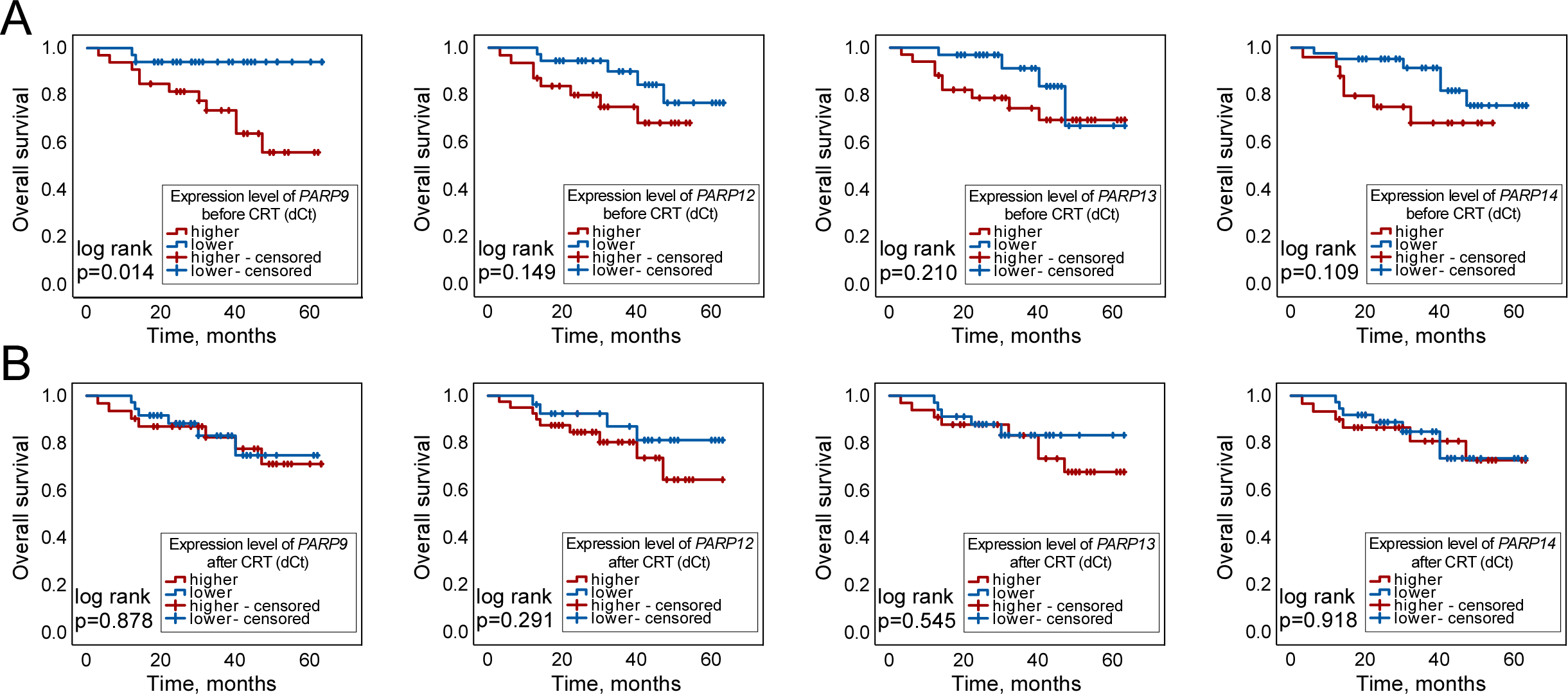


**Additional figure A14.** Kaplan-Meier survival curves demonstrating the association between expression level of PARP genes before (**A**) and after (**B**) CRT and overall survival (OS) in rectal cancer samples (n=67). Patients were stratified into high and low change of expression groups according to the mean value. Curves were compared using the log-rank test, *p* values are shown.


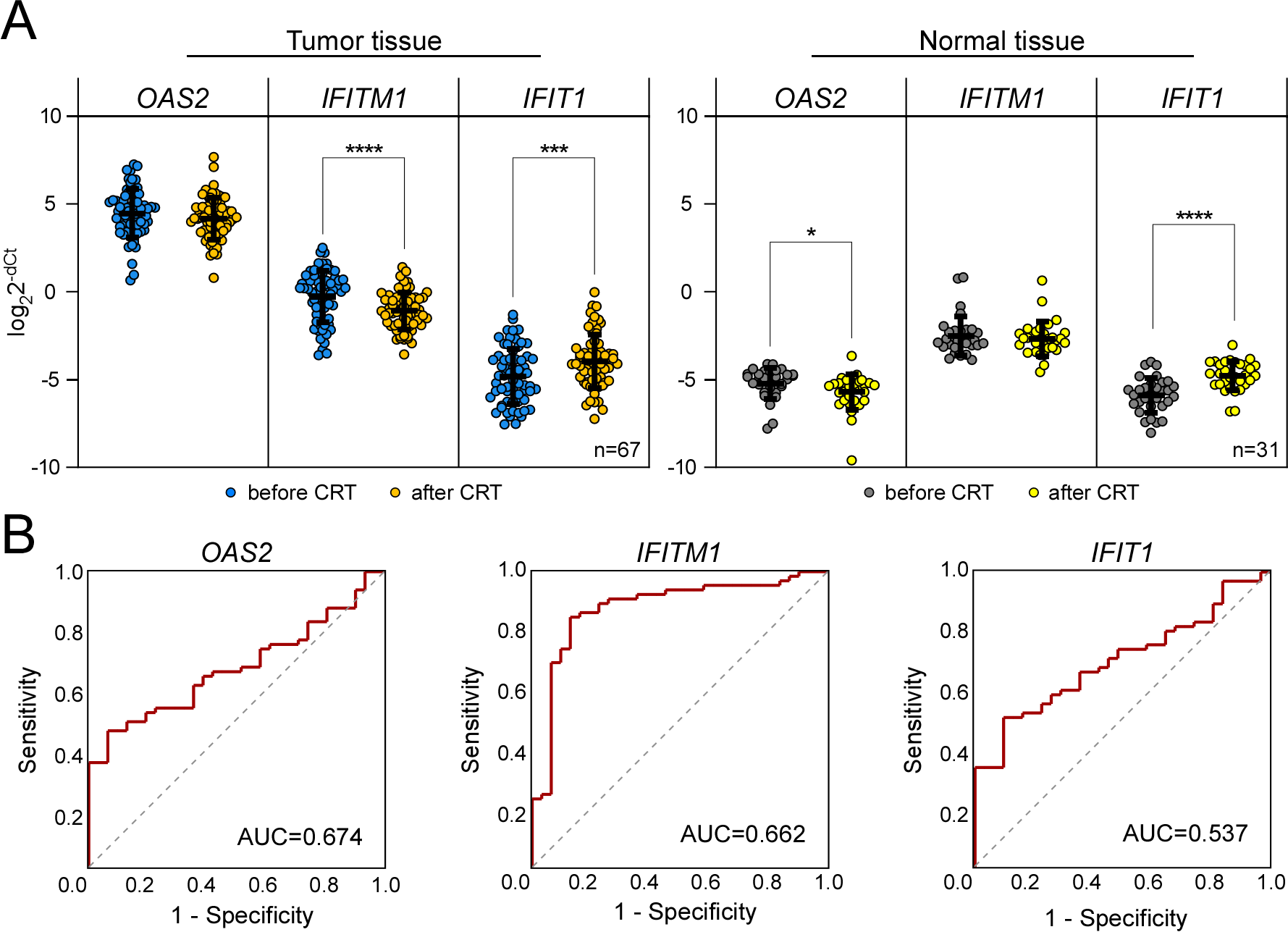


**Additional figure 15.** (**A**) qRT-PCR analysis of relative *OAS2, IFITM1*, and *IFIT1* gene expression levels before and after CRT in tumor and normal tissue sample groups. The cycle threshold (Ct) values of observed genes were normalized to *GAPDH, ACT and TBP*. Lines within boxes indicate relative gene expression mean values, while whiskers denote standard deviation of the relative gene expression values (tumor n=67, normal n=31, Student’s t test, **p*<0.05, ****p*<0.001 and *****p*<0.0001). (**B**) Diagnostic ROC curve analysis showing sensitivity and specificity of *OAS2, IFITM1,* and IFIT1 before CRT. AUC denotes the area under the ROC curve.


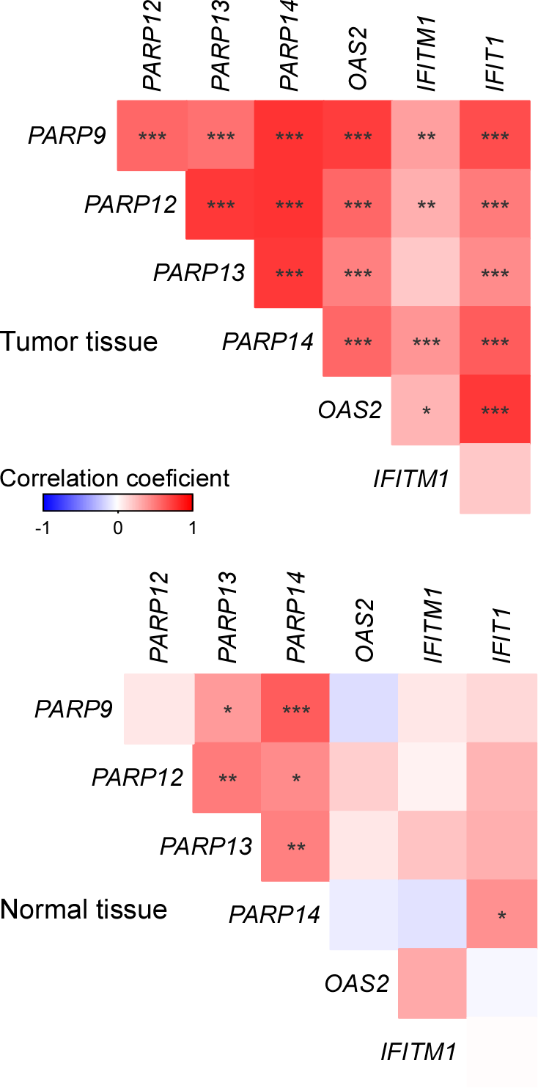


**Additional figure 16.** PARPs and selected immune response genes co-expression analysis in rectal tumor (n=67) and normal (n=31) tissue samples after CRT. Colors represent Pearson’s correlation coefficient r, **p*<0.05; ***p*<0.01; ****p*< 0.001.


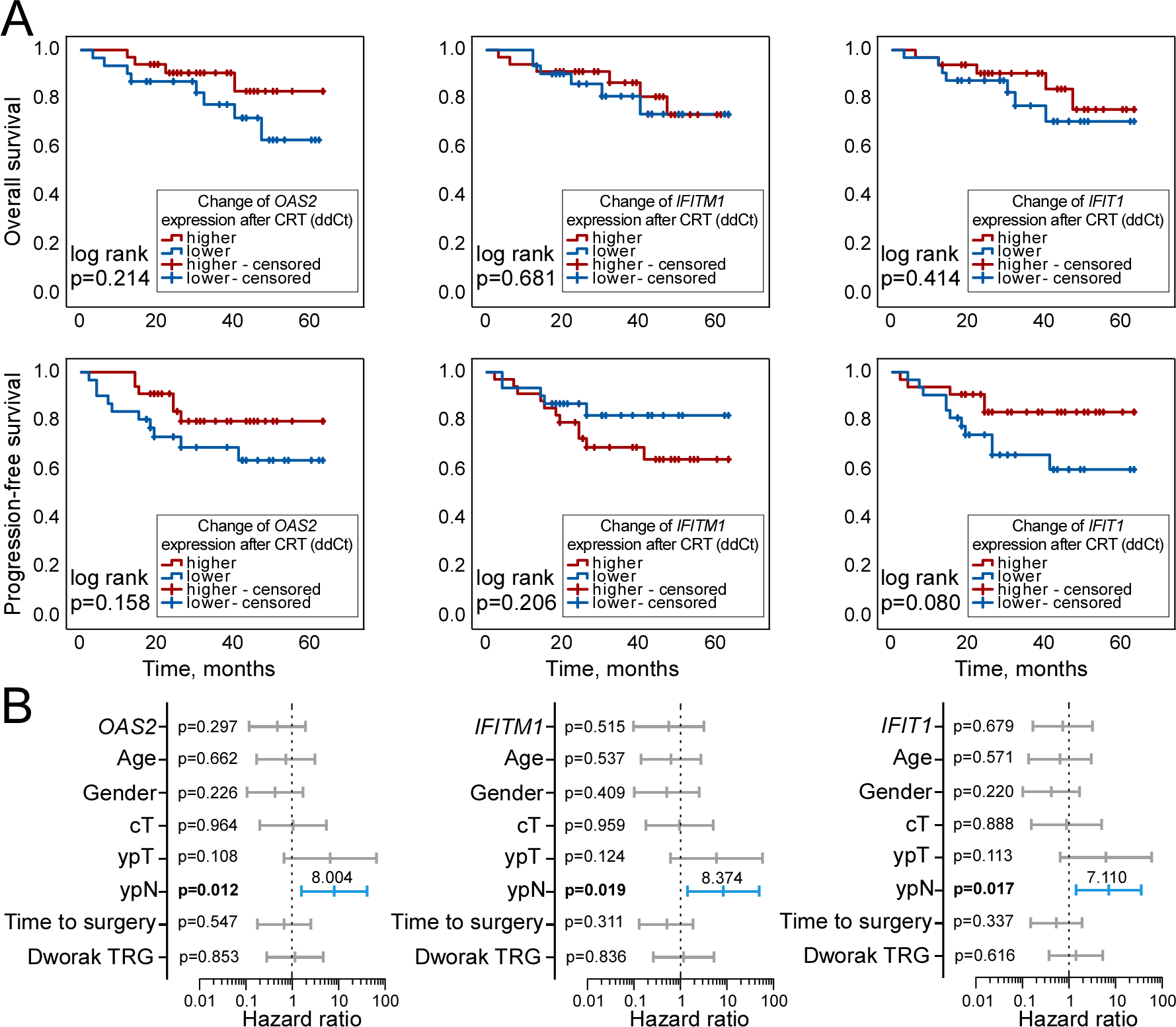


**Additional figure 17**. (**A**) Kaplan-Meier survival curves demonstrating the association between changes in *OAS2*, *IFITM1* and *IFIT1* expression after CRT and overall survival (OS) or progression-free survival (PFS) in rectal cancer tissue samples (n=67). Patients were stratified into high and low change of expression groups according to the mean value. Curves were compared using the log-rank test, *p* values shown. (**B**) Prognostic performance of *OAS2*, *IFITM1* and *IFIT1* expression changes and clinicopathologic features by multivariate Cox regression analysis. Forest plot illustrates the hazard ratio (vertical bar and number above it) and 95% confidence intervals (whiskers) associated with predictors for rectal cancer patients’ OS in tumor samples (n=67). Significant predictors are highlighted in blue, with displayed *p* values.
